# Exposure to arsenic at different life-stages and DNA methylation meta-analysis in buccal cells and leukocytes

**DOI:** 10.1186/s12940-021-00754-7

**Published:** 2021-07-09

**Authors:** Anne K. Bozack, Philippe Boileau, Linqing Wei, Alan E. Hubbard, Fenna C. M. Sillé, Catterina Ferreccio, Johanna Acevedo, Lifang Hou, Vesna Ilievski, Craig M. Steinmaus, Martyn T. Smith, Ana Navas-Acien, Mary V. Gamble, Andres Cardenas

**Affiliations:** 1 Division of Environmental Health Sciences, School of Public Health, University of California, 2121 Berkeley Way, Room 5302, Berkeley, Berkeley, CA 94720 USA; 2 Graduate Group in Biostatistics, University of California, Berkeley, Berkeley, CA USA; 3 Department of Environmental Health and Engineering, The Johns Hopkins University Bloomberg School of Public Health, Baltimore, MD USA; 4 Advanced Center for Chronic Diseases (ACCDiS), School of Medicine, Pontificia Universidad Católica de Chile, Santiago, Chile; 5 Department of Public Health, School of Medicine, Pontificia Universidad Católica de Chile, Santiago, Chile; 6 Health Planning Division in the Ministry of Health, Santiago, Chile; 7 Department of Preventive Medicine, Feinberg School of Medicine, Northwestern University, Chicago, IL USA; 8 Department of Environmental Health Science, Mailman School of Public Health, Columbia University, New York City, NY USA

**Keywords:** Arsenic, DNA methylation, Epigenetics, Prenatal exposure

## Abstract

**Background:**

Arsenic (As) exposure through drinking water is a global public health concern. Epigenetic dysregulation including changes in DNA methylation (DNAm), may be involved in arsenic toxicity. Epigenome-wide association studies (EWAS) of arsenic exposure have been restricted to single populations and comparison across EWAS has been limited by methodological differences. Leveraging data from epidemiological studies conducted in Chile and Bangladesh, we use a harmonized data processing and analysis pipeline and meta-analysis to combine results from four EWAS.

**Methods:**

DNAm was measured among adults in Chile with and without prenatal and early-life As exposure in PBMCs and buccal cells (*N* = 40, 850K array) and among men in Bangladesh with high and low As exposure in PBMCs (*N* = 32, 850K array; *N* = 48, 450K array). Linear models were used to identify differentially methylated positions (DMPs) and differentially variable positions (DVPs) adjusting for age, smoking, cell type, and sex in the Chile cohort. Probes common across EWAS were meta-analyzed using METAL, and differentially methylated and variable regions (DMRs and DVRs, respectively) were identified using comb-p. KEGG pathway analysis was used to understand biological functions of DMPs and DVPs.

**Results:**

In a meta-analysis restricted to PBMCs, we identified one DMP and 23 DVPs associated with arsenic exposure; including buccal cells, we identified 3 DMPs and 19 DVPs (FDR < 0.05). Using meta-analyzed results, we identified 11 DMRs and 11 DVRs in PBMC samples, and 16 DMRs and 19 DVRs in PBMC and buccal cell samples. One region annotated to *LRRC27* was identified as a DMR and DVR. Arsenic-associated KEGG pathways included lysosome, autophagy, and mTOR signaling, AMPK signaling, and one carbon pool by folate.

**Conclusions:**

Using a two-step process of (1) harmonized data processing and analysis and (2) meta-analysis, we leverage four DNAm datasets from two continents of individuals exposed to high levels of As prenatally and during adulthood to identify DMPs and DVPs associated with arsenic exposure. Our approach suggests that standardizing analytical pipelines can aid in identifying biological meaningful signals.

**Supplementary Information:**

The online version contains supplementary material available at 10.1186/s12940-021-00754-7.

## Background

Chronic exposure to arsenic through drinking water affects an estimated 140 million people worldwide [1]. Arsenic is a known human toxicant and carcinogen [2] associated with a range of adverse health outcomes including skin lesions, impaired intellectual function, cardiovascular disease, diabetes, inflammation, and cancers including bladder, lung, kidney, liver, and skin [2–4]. Associations between arsenic exposure and latent disease risk may be mediated by epigenetic mechanisms including dysregulation of DNA methylation (DNAm) [5, 6]. DNAm may also serve as a biomarker of past arsenic exposure and future disease risk.

In epidemiological studies, arsenic exposure has been associated with changes in global levels of DNAm [7]. Arsenic-induced changes in the DNA methylome have also been studied in epigenome-wide association studies (EWAS), which had previously been reviewed and summarized [8, 9]. EWAS most commonly measure DNAm on the individual-locus level using the Illumina HumanMethylation BeadChip (450K) or Illumina Infinium MethylationEPIC BeadChip (850K) which interrogate > 450,000 and > 850,000 methylation sites, respectively. Previous EWAS have commonly studied prenatal arsenic exposure and DNAm measured in cord blood and placenta samples among birth cohorts in the United States (US) (*N* = 136; 343) [10, 11], Bangladesh (*N* = 44; 45; 113; 127) [12–15], Mexico (*N* = 38) [16], and Taiwan (*N* = 64) [17]. Studies of exposure later in the life course have measured DNAm in blood samples, including adults with low (*N* = 46) [18] or low-to-moderate exposure in the US (*N* = 2325) [9], moderate-to-high exposure in Bangladesh (*N* = 400; 396) [19, 20], and women in Argentina (*N* = 96) [21].

Although EWAS have consistently identified CpGs associated with arsenic exposure after adjusting for multiple comparisons, a common epigenetic signature of arsenic exposure has not emerged across different studies or regions. Methodological differences could have contributed to inconsistent results and have limited comparison across EWAS. These include differences between populations (like age and ancestry), timing and level of exposure, methods for quantifying exposure and DNAm, and data processing and analysis (including normalization and adjusting for cell type distribution). Furthermore, previous EWAS have often been limited by small sample sizes, resulting in low statistical power after adjusting for multiple comparisons.

In the current study, we aim to address limitations of comparing results across EWAS and provide a model for leveraging EWAS with small sample sizes to detect epigenome-wide associations with environmental exposures. We use a two-step process of applying (1) a harmonized data processing and analysis pipeline and (2) combining results in a meta-analysis using four DNAm data datasets. Specifically, we leverage data from a cohort study in Chile with participants with and without high levels of prenatal and early-life arsenic exposure selected for DNAm measurement in peripheral blood mononuclear cells (PBMCs) and buccal cells using the 850K microarray (*N* = 40), and data from a randomized controlled trial in Bangladesh with adults classified as high or low exposure selected for DNAm measure using the 450K microarray (*N* = 48) and 850K microarray (*N* = 32). EWAS in individual studies were performed to identify differentially methylated positions (DMPs) and differentially variable positions (DVPs), and meta-analyses were performed for DMPs and DVPs. Differentially methylated regions (DMRs) and differentially variable regions (DVRs) were identified from meta-analysis results. We hypothesized that by standardizing preprocessing data pipelines and statistical methods we could increase our power to detect commons arsenic related DNAm signatures.

## Methods

### Study design

#### Chile study

For this study we selected participants residing in Antofagasta who were born in Region II of Chile (i.e., the cities/towns of Calama, Antofagasta, Chuquicamata, Maria Elena, Pedro de Valdivia, and Tocopilla), during a period of relatively high arsenic exposure between 1958 and 1972 [22]. The city level of arsenic exposure in Calama and Antofagasta at that time has been estimated to be around 287 and 860 μg/L, respectively [23]. After this period, arsenic concentrations were abruptly reduced after the installation of arsenic removal plants, initially to about 110 μg/L and continuing with improvements over the years, such that the water arsenic concentrations in Antofagasta other towns and cities in Region II was less than 100 μg/L by the late 1980s and have since been less than 10 μg/L [24, 25]. Exposed participants were born in Region II between 1958 and 1972 and were between the ages of 41–55 years old at the time of inclusion in the DNAm study. All exposed participants experienced prenatal exposure; a large proportion also experienced early-life exposure from birth to ~ 14 years of age. Unexposed participants were people born elsewhere but moved to Antofagasta when they were older, after the high exposure period.

Participants were recruited using convenience sampling following Institutional Review Board (IRB)-approved informed consent protocols among people who work at or visit the Antofagasta Hospital or the University of Antofagasta. Study protocols were approved by the University of California (UC), Berkeley and the Pontificia Universidad Católica de Chile IRBs.

This study comprises 40 participants: 20 exposed, and 20 unexposed. A sample of buccal and PBMCs were collected from each participant in 2013. Four additional samples of each tissue type were collected at random from the participants for a total of 44 samples of each tissue. Repeated donors were used as technical duplicates and were removed prior to data processing.

##### Arsenic exposure

Participants were classified as exposed if they were exposed to high levels of arsenic prenatally and in early life. Participants were primarily exposed to high levels of arsenic prenatally, although participants born at the beginning of the recruitment window were also exposed in early life. The remaining participants were designated as unexposed, given that their level of arsenic exposure was likely much lower. Volunteers were excluded for the following: antibiotic use in the 3 months prior to the study, use of enemas or laxatives more than once per month, or use of steroids or immunosuppressants.

##### Sample collection and processing

Blood samples were collected by certified nurses in Heparin-containing tubes at the participants' homes or other convenient location for the participants in Antofagasta, Chile. Ten ml of whole blood per tube was separated into plasma, buffy coat, and red blood cell fractions. Buffy coats from two collection tubes from the same participant (~ 0.5 mL each) were diluted up to 8 ml with phosphate buffered saline (PBS), and layered atop 4 ml Ficoll. After centrifugation at 380 x g for 40 min, the mononuclear layer and ~ 1 ml of the Ficoll was transferred to a new tube, diluted up to 12 ml with sterile PBS, and centrifuged for 10 min. After the wash, the supernatant was removed, and the pellet containing PBMCs was resuspended in 2 ml of PBS. PBMCs were cryopreserved with a solution of 10% dimethyl sulfoxide (DMSO) and 40% fetal bovine serum in cryotubes and placed in Coolcell cryogenic storage containers (Corning, Tewksbury, MA) at − 80 °C. After 24 h, the cryopreserved PBMCs were transferred into liquid nitrogen vapor phase until transport on dry ice to UC Berkeley. For DNA extraction, PBMCs were thawed (< 30 s) in a 37 °C water bath, diluted in 20 mL sterile PBS and centrifuged for 10 min. The supernatant was removed, and DNA was extracted from PBMC using the Allprep DNA/RNA/miRNA universal kit (Qiagen, Germantown, MD).

Buccal cells were collected by the participants by brushing a nylon ­flocked solid shaft swab (Copan Diagnostics, Murrieta, CA) against the inside of the cheek (rotating and brushing 5 times). The swab was then placed into a collection tube containing 2 ml RNALater (Thermo Scientific, Waltham, MA) and the tip was cut off with sterile scissors. Samples were stored at − 80 °C until transport on dry ice to UC Berkeley. DNA was extracted from the buccal cell sampled using the Allprep DNA/RNA/miRNA universal kit (Qiagen, Germantown, MD). PBMC and buccal cell DNA was visualized on 1% agarose gels for quality and quantified with a NanoDrop 1000 (Thermo Scientific, Waltham, MA) and Quant-iT PicoGreen dsDNA assay kit (Life Technologies, Grand Island, NY).

##### DNAm measurement

The 850K microarray (Infinium Human Methylation EPIC BeadChip microarray, Illumina, San Diego, CA) was used to measure DNAm in the Chile study. 850K analyses were conducted at the California Institute for Quantitative Biosciences (QB3) at UC Berkeley.

#### Bangladesh study

The Folic Acid and Creatine Trial (FACT) is a randomized controlled trial conducted between 2010 and 2012 to evaluate the effects of folic acid and creatine supplementation on blood arsenic concentrations and arsenic methylation capacity among arsenic-exposed adults in Araihazar, Bangladesh [26]. Briefly, participants were randomly selected from the Health Effects of Arsenic Longitudinal Study (HEALS) cohort [27] and were eligible for FACT if they were drinking from a household well with arsenic concentrations ≥ 50 μg/L for at least one year prior. Exclusion criteria were pregnancy, taking nutritional supplements, or having proteinuria, renal disease, diabetes, gastrointestinal problems, or other health issues. A total of 622 participants were recruited and randomly assigned to one of five treatment groups: 400 μg FA/day, 800 μg FA/day, 3 g creatine/day, 3 g creatine and 400 μg FA/day, and placebo.

The study was approved by the IRB at Columbia University Medical Center and the Bangladesh Medical Research Council, and participants provided informed consent.

A subset of 48 male participants was selected for DNAm analysis of blood collected at baseline using the 450K microarray in 2011. DNAm was also measured in baseline samples for an additional 32 male participants from the creatine and creatine+400FA treatment groups using the 850K microarray in 2018. Participants were selected based on the availability of a sufficient DNA sample and to represent a wide range of arsenic exposures. Due to differences in technology of the 450K and 850K platforms and timing that the two analyses were run, we chose to analyze the Bangladesh 450K and 850 K samples as independent data.

##### Arsenic exposure

Arsenic exposure was assessed using baseline water samples collected at the time of recruitment into the HEALS cohort between 2000–2002 and 2007–2008. Baseline water arsenic concentrations were measured using unfiltered samples collected from the tube well used by each participant. Water was collected in 20-mL polyethylene scintillation vials, acidified to 1% with high-purity Optima HCl (Fisher Scientific, Pittsburg, PA), and diluted 1:10. Arsenic concentrations were measured with high-resolution inductively coupled plasma mass spectrometry (ICP-MS) including a germanium spike for correcting for fluctuations in sensitivity.

##### Sample collection and processing

Blood samples were collected in EDTA-containing tubes at the field clinic in Araihazar, Bangladesh. Eight ml was separated into plasma and red blood cell fractions, the red blood cell fraction was diluted to 15 ml with PBS, and 7.5 ml was layered atop 4 ml Ficoll. After centrifugation at 400 x g for 30 min, the mononuclear layer and half of the Ficoll was transferred to a new tube and centrifuged for 10 min with an equal volume of PBS. Cells were washed with PBS, the supernatant was removed, and the pellet was resuspended in 3.5 ml 5 PRIME ArchivePure DNA Cell Lysis solution (Gaithersburg, MD) with proteinase K. Samples were stored at − 80 °C until transport on dry ice to Columbia University. DNA was extracted from PBMCs with the 5 PRIME ArchivePure DNA Blood Kit (Gaithersburg, MD) and visualized on gels for quality with the Quant-iT PicoGreen dsDNA assay kit (Life Technologies, Grand Island, NY). DNA was quantified using the Quant-iT PicoGreen dsDNA assay kit (Life Technologies, Grand Island, NY).

##### DNAm measurement

The 450K microarray (Infinium HumanMethylation450 BeadChip microarray, Illumina, San Diego, CA) or 850K microarray (Infinium Human Methylation EPIC BeadChip microarray, Illumina, San Diego, CA) was used to measure DNAm for the Bangladesh study. In the Bangladesh study, 450K analyses were conducted at Roswell Park Cancer Institute, and 850K analyses were conducted at the Center for Population Epigenetics at Northwestern University.

### Data processing

Raw idat files were imported to R using the *minfi* package [28]. Standard quality control plots were generated (i.e., density and bean plots of raw Beta-values and plots of control probe intensities) and inspected to identify low-quality samples. Outlying samples were also identified by plotting the log_2_ of the methylated and unmethylated median intensity values. One outlying sample was removed from the Chile study’s set of buccal tissue samples. All PBMC samples from the Chile and Bangladesh studies passed the quality control checks. In the Chile study, technical duplicates in buccal and PBMC samples were removed such that a total of 39 buccal samples and 40 PBMC samples were retained for analysis. In the Bangladesh study, 48 450K and 32 850K samples were used in analyses.

Detection *p*-values for each probe were calculated using the QCinfo function implemented by the *ENmix* package [29]. Probes were filtered using the champ.filter function implemented by the *ChAMP* package: probes with detection *p*-values > 0.01, non-cg, probes that align to multiple locations [30], and probes located on the X and Y chromosomes were removed [31, 32]. Functional normalization (FunNorm) was performed to mitigate technical variation [33]. The impact of known technical variables (e.g., batch, column, row) was assessed using principal component analysis (PCA) and visualizing the first five PCs. In the Bangladesh 450K study, the combat function in the *sva* package [34] was used to correct for clustering by batch. Prior to data analysis, an additional probe filter was applied to remove cross-reactive probes [35] and probes located with 10 base pairs of SNPs with minor allele frequencies ≥ 0.05 using the rmSNPandCH function in *DMRcate* [36].

For PBMC samples, the proportions of CD8+ T cells, CD4+ T cells, natural killer (NK) cells, B cells, monocytes, and neutrophils were estimated using the Houseman regression calibration method [37] with the estimateCellCounts function in *minfi* and the IlluminaHumanMethylation450k reference set. For buccal cell samples, six latent factors estimated via ReFACTor were used to control samples’ cell-type compositions in downstream analyses [38].

Data processing and analyses were conducted using R 4.0.2 [39] and packages were downloaded from Bioconductor [40]. R code for the data processing pipeline and quality control plots are available on the study’s GitHub repository [41].

### Data analysis

Descriptive statistics were calculated for each study (mean and SD for continuous variables, and frequency and proportion for categorical variables). Arsenic exposure was defined as a dichotomous variable. In the Chile study, participants were assigned to the exposure group if they were exposed to high levels of arsenic prenatally and in early life and compared to unexposed participants. For comparability and to improve chronic arsenic exposure misclassification, participants in the Bangladesh study were classified as having low or high arsenic exposure based on drinking water arsenic concentrations. The cutpoint of ≤ 100 μg/L or > 100 μg/L (i.e., the median value) was used for the 450K study. A large proportion of participants in the 850K study had water arsenic concentrations near this value (*N* = 9 with water arsenic concentrations between 100 and 104 μg/L) and therefore a cut point of ≤ 104 μg/L or > 104 μg/L was used. Associations between arsenic exposure and Houseman cell type proportions of PBMC samples were assessed using linear models adjusted for age, smoking status (ever smoker), and, in the Chile study, sex.

#### EWAS

EWAS for DMPs and DVPs associated with arsenic exposure were performed separately for PBMC and buccal samples within the Chile study, and for the 450K and 850K platforms within the Bangladesh study. M-values were calculated as the logit transformation of Beta-values to meet model assumptions. DMPs were identified using the R package *limma*, which implements linear models with a robust empirical Bayes smoothing of standard errors [42]. DVPs were identified using the varFit function in the missMethyl package [43, 44] to fit linear models on the absolute residuals for each probe. Models were adjusted for (1) age, smoking status, and sex (in the Chile study), and (2) age, smoking status, sex, and estimated cell type proportions (determined using the Houseman regression calibration method for PBMC samples [37] and ReFACTor for buccal cell samples [38]). We adjusted for multiple comparisons using the Benjamini and Hocherg false discovery rate (FDR) correction [45] and the Bonferroni correction, and adjusted *p*-values were calculated using the p.adjust function.

#### Meta-analyses

EWAS results were meta-analyzed using METAL [46]. METAL calculates pooled effect sizes for each probe by weighting the effects of individual EWAS by the inverse of the standard errors and calculates overall Z-scores and *p*-values. We conducted meta-analyses for DMPs and DVPs using (1) PBMC samples only (i.e., Chile study PBMC EWAS, Bangladesh study 450K EWAS, and Bangladesh study 850K EWAS; referred to as PBMC meta-analysis) and (2) all PBMC samples and the Chile buccal cell EWAS (referred to as PBMC + buccal cell meta-analysis). Analyses were restricted to probes common across the four studies (377,351 CpG probes). Sensitivity analyses were conducted by performing meta-analyses on EWAS results not adjusted for estimated cell type proportions. The genomic inflation factor (λ) and Q-Q plots were used to evaluate systematic biases in meta-analysis results. Heterogeneity was assessed by calculating I^2^ and Cochran’s Q-test [47, 48].

Given that METAL assumes the individual EWASs to be independent, we verified that the Chile study PBMC and buccal cell samples could indeed be treated as such. As expected, we observed the major source of variation among the Chile samples to be tissue type, detecting no evidence of a strong within-participant effect when performing PCA on the M-Values of the 5000 most variable CpG sites (Supplemental Fig. S1). Further, we computed the similarity between each participants’ processed PBMC and buccal cell samples’ M-values using the Spearman correlation, excluding the individual whose buccal cell sample did not pass quality control checks. We then computed the similarity scores of all possible combinations of independent PBMC and buccal cell samples, generating the null distribution of scores under the assumption of independent sample origin. The observed within-participant similarities were not found to be significant after correcting for multiple testing (*FDR* < 0.05). Only one participant’s samples were more similar than expected under the null at the nominal level of significance (*p* < 0.05) (Supplemental Fig. S2).

We analyzed meta-analyses’ results for DMRs with respect to DMPs and DVPs using comb-p [49]. The following criteria were used to define a region: the leading probe had a *p*-value of 0.001 or smaller, and the region contained no less than three probes. Regions had a minimum length of 1000 base pairs (bp), and, if another probe within the region satisfied the significance cutoff, were extended by 1000 bp starting from said probe.

Pathway analyses were conducted to better understand the biological functions of DMPs and DVPs identified in meta-analyses. Pathway analysis with the Kyoto Encyclopedia of Genes and Genomes (KEGG) database was conducting using the gometh function [50] implemented by the *missMethyl* R package [43]. Gometh is adapted for studies using the 450K or 850K microarrays to account for the a priori probability of a gene to include a DMP or DVP based on representation on the array. DMPs and DVPs with *FDR* < 0.10 in METAL results were used as input for gometh.

#### Comparison to previous EWAS

To assess the consistency of our results with those of previous studies, we searched the Comparative Toxicogenomics Database [51] for interactions between arsenic and the genes containing DMPs and DVPs. We also compared our identified DMPs and DVPs with nominally significant DMPs reported by Bozack et al. [9] and DMPs significant after adjustment for multiple comparisons reported by EWAS of arsenic exposure [10–21, 52, 53].

## Results

Participant characteristics for the four studies are presented in Table 1. In the Chile PBMC and buccal cell studies, 50 and 49% of participants were exposed to arsenic prenatally or in early life, respectively. In the Bangladesh 450K study and 850K studies, 23 (48%) and 11 (34%) of participants were exposed to high arsenic, respectively (≥ 100 μg/L water arsenic and ≥ 104 μg/L water arsenic). The median (range) of water arsenic for low and high exposure groups in the 450K study were 57.0 μg/L (50.0–100.0 μg/L) and 249.7 (160.0–500.0 μg/L), respectively; the median (range) for low and high exposure groups in the 850K study were 94.0 μg/L (50.0–104.0 μg/L) and 250.0 μg/L (112.0–500.0 μg/L), respectively. Prenatal exposure to arsenic in Bangladesh was unknown. The mean (SD) age for the Chile studies was 48.7 (4.7) years, whereas the mean (SD) ages for the Bangladesh 450K and 850K studies were slightly lower 39.7 (8.1), and 41.1 (6.3) years. Approximately half participants in the Chile studies were male; all participants in the Bangladesh studies were male.

**Table 1:** Participant characteristics

	Chile study, PBMCs (***N*** = 40) ^a^		Chile study, buccal cells (***N*** = 39) ^a^		Bangladesh study, 450 K (***N*** = 48) ^b^		Bangladesh study, 850 K (***N*** = 32) ^b^	
	n	%	n	%	n	%	n	%
Age, years, mean (SD)	48.7	(4.7)	48.7	(4.7)	39.7	(8.1)	41.7	(6.3)
Male	21	52.5	20	51.3	48	100.0%	32	100.0%
Ever smoker	16	40.0	16	41.0	21	43.8%	20	62.5%
Prenatal/early-life arsenic exposure	20	50.0	19	48.7	–	–	–	–
High arsenic exposure ^c^	–	–	–	–	23	47.9%	11	34.4%

^a^ DNAm analysis of PBMC and buccal cell samples performed among the same study participants. ^b^ DNAm analyses in Bangladesh studies conducted in PBMCs. ^c^ Cutoff of ≥ 100 μg/L water arsenic used to classify arsenic exposure for 450K analyses (low exposure median, range, and IQR: 57.0 μg/L; 50.0–100.0 μg/L; 51.5, 72.9 μg/L; high exposure median, range, and IQR: 249.7 μg/L; 160.0–500.0 μg/L; 173.5, 253.5 μg/L) and 104 μg/L water arsenic used to classify arsenic exposure for 850K analyses (low exposure median, range, and IQR: 94.0 μg/L; 50.0–104.0 μg/L; 67.0, 100.0 μg/L; high exposure median, range, and IQR: 250.0 μg/L; 112.0–500.0 μg/L; 143.0, 363.5 μg/L)

Associations between arsenic exposure and Houseman cell type proportion estimates in PBMC samples are shown in Table 2. Arsenic exposure was positively associated with the proportion of CD8+ T cells (*B* = 0.05; *p* = 0.018) and negatively associated with the proportion of monocytes (*B* = − 0.05, *p* = 0.003) in the Bangladesh 450K study; however, we did not observe significant associations between arsenic exposure and cell type proportions in either of the other studies.

**Table 2:** Associations between arsenic exposure and cell type proportion estimates

	Chile study, PBMCs (N = 40)		Bangladesh study, 450 K (N = 48)		Bangladesh study, 850 K (N = 32)	
	***B***^a^	***p***	***B***^a^	***p***	***B***^a^	***p***
CD8+ T cells	−0.03	0.18	0.05	0.018	0.02	0.28
CD4+ T cells	−0.02	0.54	0.00	0.93	0.01	0.66
NK	0.01	0.89	0.01	0.75	−0.06	0.14
B cells	0.03	0.11	0.02	0.16	0.02	0.42
Monocytes	0.01	0.58	−0.05	0.003	− 0.01	0.73
Granulocytes	0.00	0.89	−0.03	0.12	0.01	0.45

^a^ Linear models predicting Housman estimated cell type proportions and adjusted by age, smoking status (ever smoker) and sex (in Chile study)

The number of DMPs identified in the individual EWAS are shown in Table 3; nominally significant DMPs in analyses adjusted for age, smoking status, cell type proportions, and sex (in the Chile studies) (*p* < 0.05) are available on the study’s GitHub repository [41]. With the exception of the Bangladesh 450K study, we identified a greater number of nominally significant DMPs after adjustment for cell type. Across EWAS, no DMPs remained significant after adjusting for multiple comparisons.

**Table 3:** Summary of results of individual EWAS. Number of differentially methylated probes (DMPs) and differentially variable probes (DVPs) identified in individual EWAS at *p* < 0.05 and *FDR* < 0.05, where applicable

	Adjusted for sex, age, and smoking status				Adjusted for cell type proportions, age, and smoking status			
	All probes		Common probes ^a^		All probes		Common probes ^a^	
**DMPs**	***p*** **< 0.05**		***p*** **< 0.05**		***p*** **< 0.05**		***p*** **< 0.05**	
Chile, PBMCs	24,853		13,558		46,946		23,116	
Chile, buccal cells	21,745		11,896		42,563		21,336	
Bangladesh, 450 K	51,839		47,876		19,913		18,301	
Bangladesh, 850 K	10,422		5919		14,935		7954	
**DVPs**	***p*** **< 0.05**	***FDR*** **< 0.05**	***p*** **< 0.05**	***FDR*** **< 0.05**	***p*** **< 0.05**	***FDR*** **< 0.05**	***p*** **< 0.05**	***FDR*** **< 0.05**
Chile, PBMCs	34,906	4	19,094	3	43,813	5	23,487	3
Chile buccal, cells	36,131	5	20,659	2	35,928	7	20,735	4
Bangladesh, 450 K	22,741	5	20,925	4	18,318	3	16,904	2
Bangladesh, 850 K	33,921	47	17,646	17	53,875	51	26,155	24

*DMP* differentially methylated position, *DVP* differentially variable position. ^a^ Limiting probes to 377,351 included in all four EWAS

A summary of DVPs identified in the individual EWAS is also provided in Table 3, and nominally significant cell type-adjusted analyses are included in the study’s GitHub repository [41]. In models adjusted for age, smoking status, cell type proportions, and sex (in the Chile studies), after correcting for multiple comparisons, we identified 5 DVPs in the Chile PBMC study, 7 DVPs in the Chile buccal cell study, 3 DVPs in the Bangladesh 450K study, and 51 DVPs in the Bangladesh 850K study (*FDR* < 0.05).

**Fig. 1:**
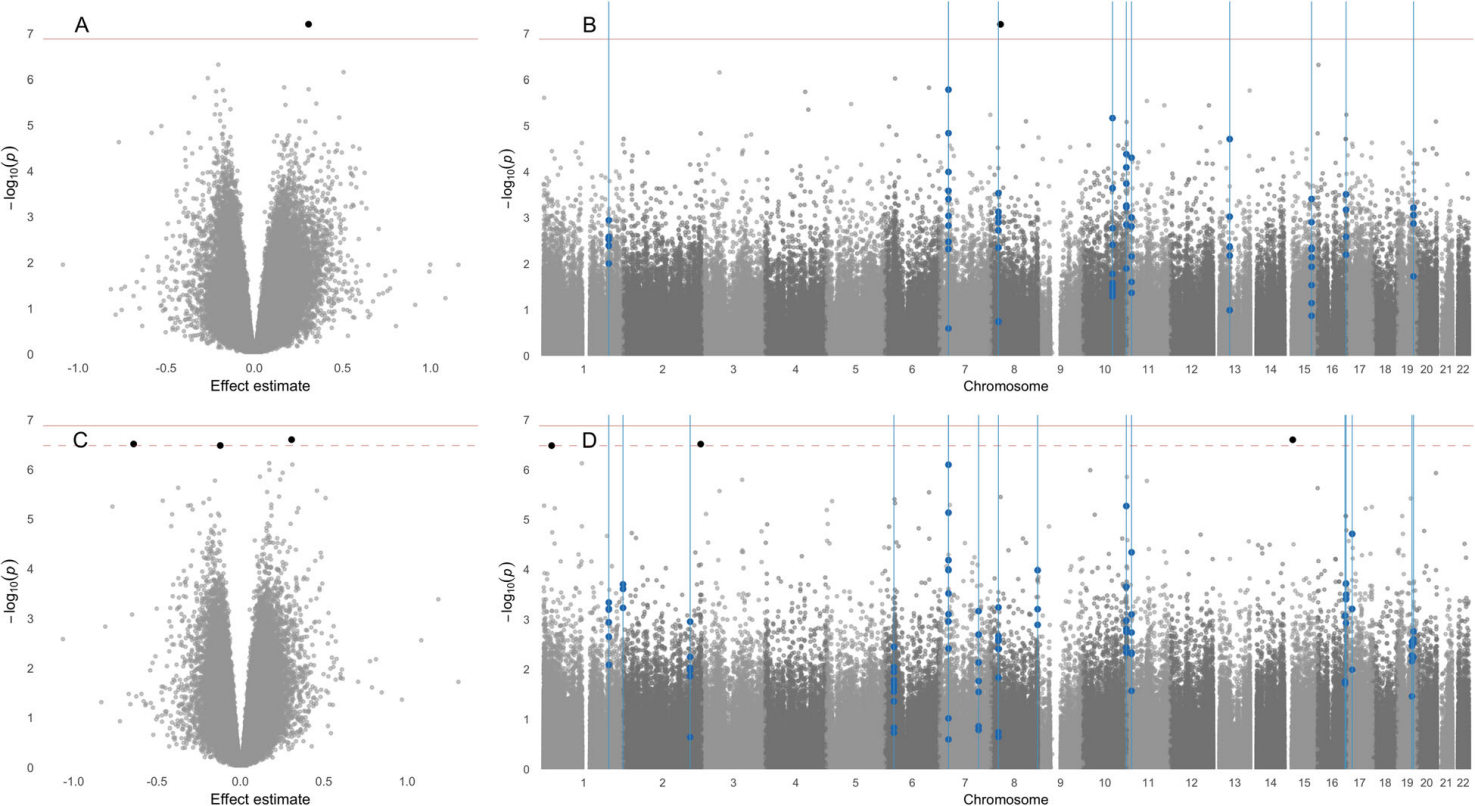
Volcano and Manhattan plots of differentially methylated positions (DMPs) and differentially methylated regions (DMRs). **A** and **B**. Volcano plot and Manhattan plot of meta-analysis of PBMC EWAS (i.e., the Chile PBMC study, Bangladesh 450K study, and Bangladesh 850K study) adjusted for age, smoking status, cell type proportions, and sex (in the Chile studies). **C** and **D**. Volcano plot and Manhattan plot of meta-analysis of PBMC + buccal cell EWAS (i.e., the Chile PBMC study, Chile buccal cell study, Bangladesh 450K study, and Bangladesh 850K study) adjusted for age, smoking status, cell type proportions, and sex (in the Chile studies). In all plots, DMPs at *FDR* < 0.05 are shown as black points; the Bonferroni and FDR levels of significance are indicated by a solid and dashed red line, respectively; and DMRs are indicated by blue lines

A summary of meta-analysis results for differential methylation and the genomic inflation factor values (λ) is provided in Table 4. Significant probes are shown in Table 5; measures of heterogeneity are included in Supplemental Table S1 and effects sizes and *p*-values of each DMP in individual EWAS are included in Additional File 1. We first conducted a meta-analysis of the three PBMC EWAS (i.e., the Chile PBMC study, Bangladesh 450K study, and Bangladesh 850K study). Using fully adjusted models including age, smoking status, cell type proportions, and sex (in the Chile studies), λ = 1.07, suggesting that our meta-analysis was not impacted by genomic inflation (Table 4 and Supplemental Fig. S3). We identified one DMP (cg13490635, annotated to *RBPMS*; *FDR* = 0.024) (Fig. 1A and B). The probe was positively associated with arsenic exposure across all EWAS but only achieved significance individually in the Bangladesh 450K study (*p* < 0.001) (Table 5 and Additional File 1), and showed evidence of moderate heterogeneity (*I*^*2*^ = 43.6; *p*_*heterogeneity*_ = 0.17) (Supplemental Table S1).

**Table 4:** Summary of results of meta-analyses. Number of differentially methylated probes (DMPs) and differentially variable probes (DVPs) and values of the genomic inflation factor (λ) in the PBMC meta-analysis (i.e., including the Chile PBMC study, Bangladesh 450 K study, and Bangladesh 850 K study) and PBMC + buccal cell meta-analysis (i.e., including the Chile buccal cell study)

	Adjusted for sex, age, and smoking status			Adjusted for cell type proportions, age, and smoking status		
	***p*** < 0.05	***FDR*** < 0.05	λ	***p*** < 0.05	***FDR*** < 0.05	λ
**DMPs**						
PBMCs	19,632	0	0.98	23,361	1	1.07
PBMCs + buccal cells	21,321	0	1.04	22,612	3	1.06
**DVPs**						
PBMCs	26,431	25	1.13	28,578	23	1.17
PBMCs + buccal cells	24,912	20	1.11	28,399	19	1.18

*DMP* differentially methylated position, *DVP* differentially variable position

**Table 5:** Differentially methylated positions (DMPs) and differentially variable positions (DVPs) in meta-analyses. DMPs and DVPs (*FDR* < 0.05) in the PBMC meta-analysis (i.e., including the Chile PBMC study, Bangladesh 450 K study, and Bangladesh 850 K study) and PBMC + buccal cell meta-analysis (i.e., including the Chile buccal cell study) adjusted for age, smoking status, cell type proportions, and sex (in the Chile studies). CpGs identified in multiple meta-analyses are bolded

CpG	Effect	Direction ^a^	***p***	***FDR***	Chr	Position	Gene	Feature category
**DMPs**								
**PBMCs**								
cg13490635	0.31	+ + +	6.30e-08	0.024	8	30,242,021	*RBPMS;RBPMS*	5’UTR;1stExon
**PBMCs + buccal cells**								
cg09275980	0.31	+ + + +	2.52e-07	0.042	15	25,350,925	*SNORD116–29*	TSS1500
cg20784693	−0.64	----	3.08e-07	0.042	2	239,984,030	*HDAC4*	Body
cg18263451	−0.12	- - + −	3.31e-07	0.042	1	25,573,180	*C1orf63*	Body
**DVPs**								
**PBMCs**								
**cg23281729**	0.97	- + +	2.86E-11	1.08E-05	18	78,005,477	*PARD6G*	TSS200
**cg27425262**	0.82	+ + +	2.26E-08	0.004	2	113,953,981	*PSD4;LOC440839*	Body;Body
**cg11361658**	0.17	+ + +	6.94E-08	0.009	5	128,452,311		
**cg03205258**	0.69	+ + +	9.72E-08	0.009	22	19,929,274	*TXNRD2;COMT; COMT*	1stExon;1stExon; 5’UTR
**cg09692492**	−0.35	---	1.36E-07	0.010	22	23,744,717	*ZDHHC8P*	Body
**cg11032634**	0.77	+ + +	3.06E-07	0.019	22	19,929,254	*TXNRD2;COMT*	1stExon;TSS200
**cg16511076**	0.13	+ + +	4.07E-07	0.022	4	48,887,480	*OCIAD2*	3’UTR
**cg22387890**	0.55	+ + +	5.44E-07	0.023	7	6,145,595	*USP42*	5’UTR
**cg22749736**	0.13	+ + +	5.53E-07	0.023	11	1,306,759	*TOLLIP*	Body
**cg04080724**	0.14	+ + +	6.06E-07	0.023	4	140,202,327	*C4orf49*	TSS1500
**cg05169951**	0.23	+ + +	6.99E-07	0.024	16	3,017,955	*KREMEN2;PAQR4*	Body;TSS1500
cg00668103	0.14	+ + +	8.08E-07	0.025	7	100,084,730	*C7orf51*	Body
cg08207566	0.12	+ + +	1.04E-06	0.030	4	174,086,461		
cg01101647	0.13	+ + +	1.13E-06	0.030	4	66,535,732	*EPHA5*	TSS200
cg07611790	0.11	+ + +	1.25E-06	0.031	19	49,240,823	*RASIP1*	Body
cg11691429	0.21	+ + +	1.39E-06	0.031	2	947,634	*SNTG2*	Body
**cg21185289**	0.38	+ + +	1.45E-06	0.031	2	74,743,437	*TLX2*	3’UTR
cg08285768	0.11	+ + +	1.48E-06	0.031	15	86,038,622	*AKAP13*	Body
cg13972353	0.15	+ + +	1.57E-06	0.031	9	139,354,348	*SEC16A*	Body
cg06740986	0.11	+ + +	2.08E-06	0.039	1	184,838,417	*FAM129A*	Body
cg21857098	0.12	+ + +	2.46E-06	0.043	15	25,638,137	*UBE3A*	Body
cg03479942	0.13	+ + +	2.50E-06	0.043	16	69,341,763	*SNTB2*	3’UTR
cg07984508	0.11	+ + +	2.73E-06	0.045	15	77,426,719	*SGK269*	Body
**PBMCs + buccal cells**								
**cg23281729**	0.99	−+++	2.00E-14	7.51E-09	18	78,005,477	*PARD6G*	TSS200
**cg27425262**	0.90	+ + + +	4.52E-13	8.53E-08	2	113,953,981	*PSD4;LOC440839*	Body;Body
**cg03205258**	0.64	+ + + +	7.95E-09	0.001	22	19,929,274	*TXNRD2;COMT; COMT*	1stExon;1stExon; 5’UTR
**cg11032634**	0.70	+ + + +	5.69E-08	0.006	22	19,929,254	*TXNRD2;COMT*	1stExon;TSS200
**cg04080724**	0.14	+ + + +	1.14E-07	0.009	4	140,202,327	*C4orf49*	TSS1500
cg26384602	−0.12	----	1.87E-07	0.010	19	41,798,386	*HNRNPUL1*	Body
**cg11361658**	0.16	+ + + +	1.90E-07	0.010	5	128,452,311		
**cg09692492**	−0.30	----	2.32E-07	0.011	22	23,744,717	*ZDHHC8P*	Body
**cg22749736**	0.12	+ + + +	4.20E-07	0.018	11	1,306,759	*TOLLIP*	Body
cg21921619	−0.15	-+--	5.48E-07	0.021	10	116,852,791	*ATRNL1*	TSS1500
cg07935287	−0.13	----	7.91E-07	0.026	18	54,318,347	*WDR7*	TSS1500
**cg05169951**	0.22	+ + + +	8.19E-07	0.026	16	3,017,955	*KREMEN2;PAQR4*	Body;TSS1500
**cg21185289**	0.37	+ + + +	1.01E-06	0.030	2	74,743,437	*TLX2*	3’UTR
**cg22387890**	0.48	+ + + +	1.12E-06	0.030	7	6,145,595	*USP42*	5’UTR
cg13171197	0.12	+ − + +	1.46E-06	0.037	7	90,916,020		
cg24754507	−0.10	----	1.55E-06	0.037	6	28,481,862	*GPX6*	Body
cg00355447	0.14	+ + + +	1.80E-06	0.039	6	30,292,220	*HCG18*	Body
**cg16511076**	0.12	+ + + −	1.87E-06	0.039	4	48,887,480	*OCIAD2*	3’UTR
cg04873963	−0.10	----	2.46E-06	0.049	16	2,518,322		

*DMP* differentially methylated position, *DVP* differentially variable position. ^a^ Direction of association between arsenic exposure and DNAm in each EWAS. For PBMCs, associations are listed in the following order: Bangladesh 850 K, Bangladesh 450 K, Chile PBMCs. For PBMCs + buccal cells, associations are listed in the following order Bangladesh 850 K, Bangladesh 450 K, Chile PBMCs, Chile buccal cells

We meta-analyzed results including the PBMC EWAS and the Chile buccal cell EWAS. Using fully adjusted models, λ = 1.06 (Table 4). We identified three DMPs: cg09275980 annotated to *SNORD116–29*; cg20784693, annotated to *HDAC4*, and cg18263451, annotated to *C1orf63* (*FDR* < 0.05) (Table 5 and Fig. 1C and D). Methylation of cg09275980 had a positive effect estimate and methylation of cg20784693 had a negative effect estimate across all four EWAS (Additional File 1). Methylation of cg18263451 was negatively associated with arsenic exposure in the Bangladesh studies and the Chile buccal cell study; the direction of association was positive in the Chile PBMC study but did not achieve statistical significance (*p* > 0.05). The probes cg09275980 and cg18263451 displayed heterogeneity across EWAS (*I*^*2*^ = 68.4; *p*_*heterogeneity*_ = 0.023 and *I*^*2*^ = 77.3; *p*_*heterogeneity*_ = 0.004, respectively) whereas there was no evidence of heterogeneity for cg20784693 (*I*^*2*^ = 0.0; *p*_*heterogeneity*_ = 0.47) (Supplemental Table S1).

In regional analysis of the PBMC meta-analysis, 11 DMRs were identified (Table 6 and Fig. 1B), and in analysis of the PBMC + buccal cell meta-analysis, 16 DMRs were identified (Table 6 and Fig. 1D). Eight DMRs overlapped between the PBMC and PBMC + buccal cell analyses. In addition, both analyses identified two DMRs within < 7 kilobase pairs located on chromosome 7 and annotated to *HOXA13* and *HOXA11-AS*.

**Table 6:** Differentially methylated regions (DMRs) and differentially variable regions (DVRs) in meta-analyses. DMRs and DVRs in the PBMC meta-analysis (i.e., including the Chile PBMC study, Bangladesh 450 K study, and Bangladesh 850 K study) and PBMC + buccal cell meta-analysis (i.e., including the Chile buccal cell study) adjusted for age, smoking status, cell type proportions, and sex (in the Chile studies). Genes identified in multiple meta-analyses are bolded

Chromosome	Start	End	N probes	Sidak ***p***	Gene
**DMRs**					
**PBMCs**					
10	134,150,451	134,150,760	7	1.47E-09	***LRRC27***
7	27,231,819	27,232,150	3	1.20E-07	***HOXA13***
10	91,295,045	91,295,650	11	1.35E-07	*SLC16A12*
7	27,225,733	27,226,148	7	8.99E-07	***HOXA11-AS***
8	23,563,925	23,564,294	8	1.26E-05	***NKX2–6***
1	203,320,190	203,320,541	6	1.35E-05	***FMOD***
11	15,095,017	15,095,178	6	5.68E-05	***CALCB***
16	88,747,591	88,747,919	4	7.15E-05	***SNAI3-AS1;SNAI3***
15	83,953,690	83,953,883	10	3.77E-05	*BNC1*
13	51,640,142	51,640,442	5	1.15E-04	*GUCY1B2*
19	49,220,102	49,220,235	4	5.36E-04	***MAMSTR***
**PBMCs + buccal cells**					
10	134,150,451	134,150,760	7	3.42E-10	***LRRC27***
7	27,225,543	27,226,148	8	4.17E-09	***HOXA11-AS***
7	27,231,819	27,232,150	3	1.04E-08	***HOXA13***
1	203,320,190	203,320,541	6	3.72E-08	***FMOD***
11	15,095,017	15,095,178	6	9.60E-07	***CALCB***
16	88,747,591	88,747,919	4	3.93E-07	***SNAI3-AS1;SNAI3***
8	23,563,925	23,564,294	8	2.69E-05	***NKX2–6***
2	207,139,131	207,139,445	6	0.001	*ZDBF2*
1	247,537,159	247,537,369	3	1.75E-05	*LOC107985115*
8	145,638,881	145,639,181	3	3.34E-05	*SLC39A4*
16	85,518,901	85,519,061	3	0.073	*GSE1*
17	17,603,837	17,604,184	3	5.40E-05	*RAI1*
6	28,584,003	28,584,155	10	0.001	*ZBED9*
19	49,220,102	49,220,235	4	5.36E-04	***MAMSTR***
7	120,968,877	120,969,174	8	1.20E-04	*WNT16*
19	44,488,121	44,488,269	5	9.10E-04	*ZNF155*
**DVRs**					
**PBMCs**					
22	19,929,066	19,929,557	9	4.27E-13	***TXNRD2;COMT;TXNRD2***
16	1,844,927	1,845,113	6	3.56E-11	***IGFALS***
22	39,784,481	39,784,982	5	7.84E-09	***SYNGR1***
3	8,809,306	8,809,715	4	6.66E-08	***OXTR***
2	947,515	947,634	3	6.36E-07	***SNTG2***
2	48,844,728	48,844,984	6	5.84E-06	***STON1-GTF2A1L;GTF2A1L***
3	48,700,269	48,700,498	8	2.02E-05	*CELSR3*
12	9,217,529	9,217,859	7	4.45E-05	***LINC00612;A2M-AS1***
16	53,407,423	53,407,808	5	7.37E-05	***LOC102723373***
10	134,150,451	134,150,760	7	6.52E-05	***LRRC27***
6	90,597,340	90,597,591	4	1.20E-04	*GJA10*
**PBMCs + buccal cells**					
22	19,929,066	19,929,557	9	5.12E-13	***TXNRD2;COMT;TXNRD2***
6	30,038,882	30,039,600	25	1.49E-09	*RNF39*
6	32,064,656	32,065,043	16	4.68E-06	*TNXB*
3	8,809,306	8,809,715	4	1.90E-07	***OXTR***
5	178,986,291	178,986,728	6	1.16E-05	*RUFY1*
6	31,651,070	31,651,291	5	0.079	*LY6G5C*
22	39,784,769	39,784,982	3	1.57E-05	***SYNGR1***
2	48,844,728	48,844,984	6	1.45E-05	***STON1-GTF2A1L;GTF2A1L***
6	32,551,749	32,552,042	3	1.72E-05	*HLA-DRB1*
12	9,217,529	9,217,859	7	4.29E-05	***LINC00612;A2M-AS1***
2	947,515	947,634	3	7.33E-05	***SNTG2***
18	59,221,320	59,221,601	3	3.20E-05	*CDH20*
16	1,844,927	1,845,113	6	6.80E-05	***IGFALS***
6	168,106,055	168,106,326	4	5.79E-05	*LINC02487*
16	53,407,423	53,407,753	4	5.41E-04	***LOC102723373***
7	157,667,762	157,668,051	4	8.85E-05	*PTPRN2*
10	134,150,451	134,150,760	7	8.96E-05	***LRRC27***
19	37,825,307	37,825,446	6	4.27E-04	*ZNF875*
19	36,246,816	36,246,906	3	8.87E-04	*HSPB6*

*DMP* differentially methylated position, *DVP* differentially variable position

**Fig. 2:**
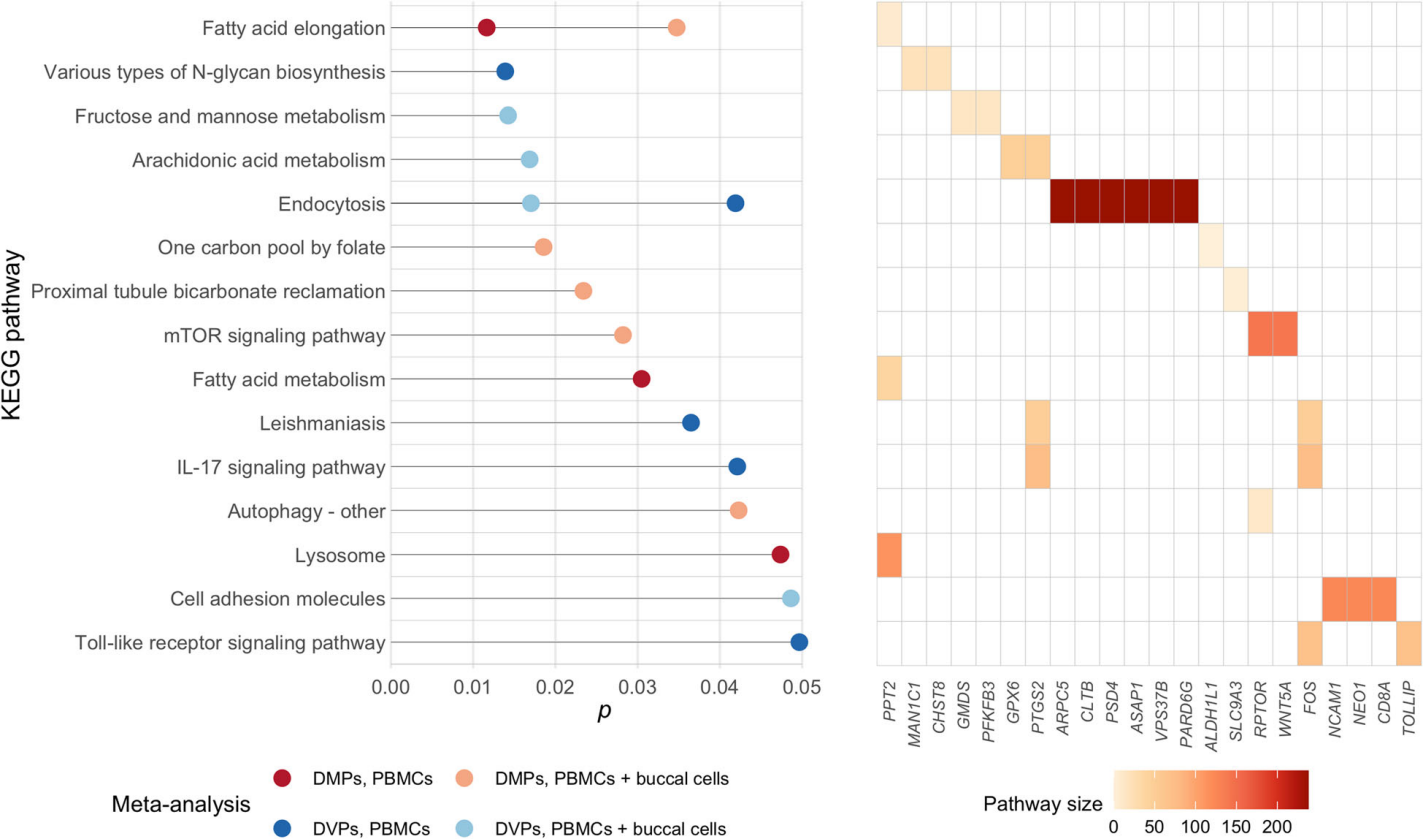
Summary of results of KEGG pathway analyses. **Left panel:** KEGG pathways (*p* < 0.05) associated with differentially methylated positions (DMPs) and differentially variable positions (DVPs) identified in the PBMC meta-analysis (i.e., including the Chile PBMC study, Bangladesh 450 K study, and Bangladesh 850 K study) and PBMC + buccal cell meta-analysis (i.e., including the Chile buccal cell study) adjusted for age, smoking status, cell type proportions, and sex (in the Chile studies). *P*-values are shown on the x-axis. **Right panel:** Genes including DMPs or DVPs that are members of each KEGG pathway

In the fully adjusted PBMC meta-analysis, 8 probes were differentially methylated at *FDR* < 0.10 and included in a KEGG pathway analysis. Three KEGG pathways were identified as including an overrepresentation of genes containing differential methylation: fatty acid elongation (*p* = 0.012), fatty acid metabolism (*p* = 0.030), and lysosome (*p* = 0.047) (Supplemental Table S2). Basal cell carcinoma and melanogenesis were among the top significant KEGG pathways but did not achieve statistical significance (*p* = 0.053 and *p* = 0.073, respectively). The pathway analysis for PBMCs + buccal cells included 25 probes (*FDR* < 0.10) and identified 5 pathways: one carbon pool by folate *(p* = 0.019), proximal tubule bicarbonate reclamation (*p* = 0.023), mTOR signaling pathway (*p* = 0.029), fatty acid elongation (*p* = 0.035), and autophagy (*p* = 0.042). No KEGG pathways achieved statistical significance after an FDR correction. Figure 2 provides the *p*-values and differentially methylated genes that overlap with each KEGG pathway identified.

**Fig. 3:**
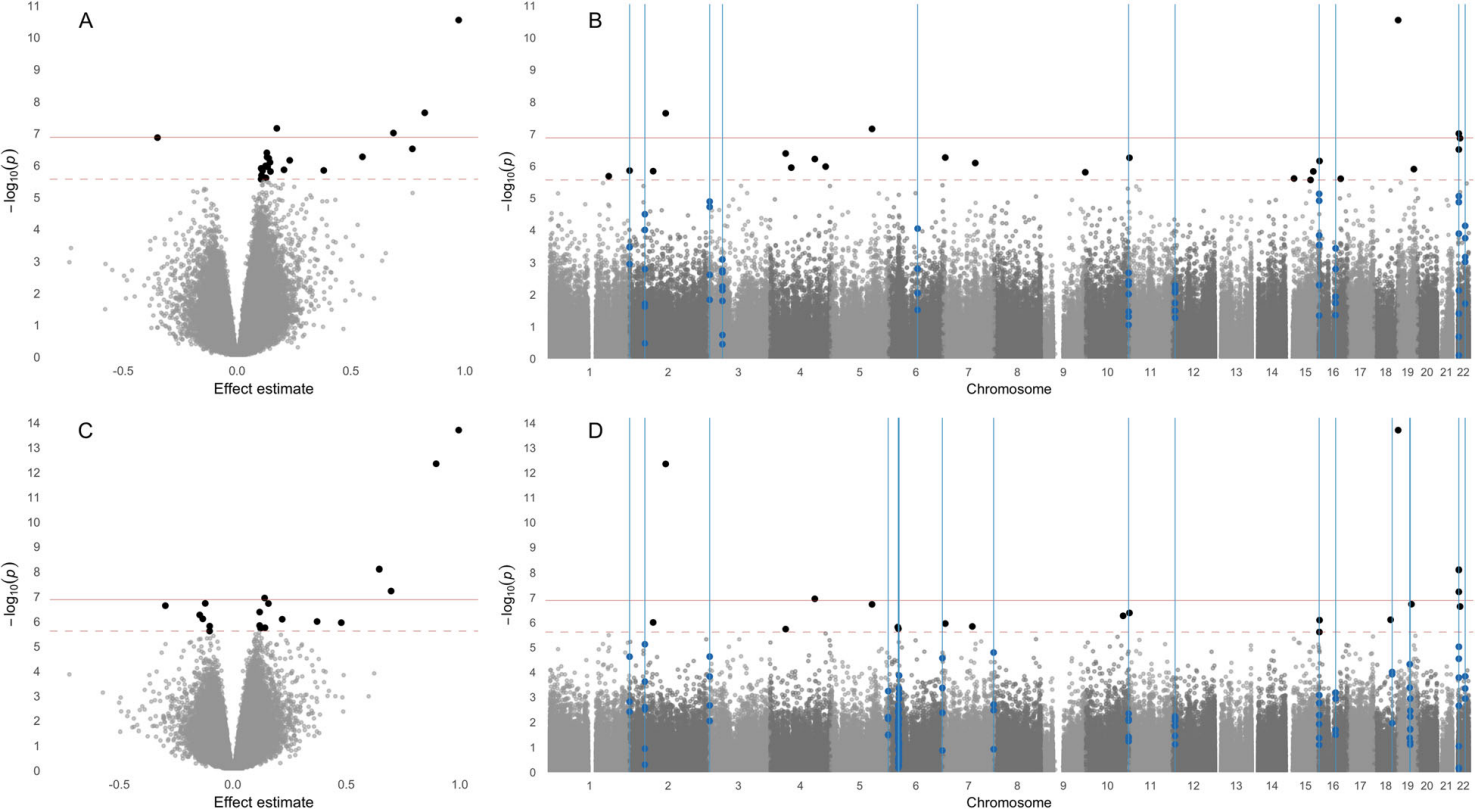
Volcano and Manhattan plots of differentially variable positions (DVPs) and differentially variable regions (DVRs). **A** and **B**. Volcano plot and Manhattan plot of meta-analysis of PBMC EWAS (i.e., the Chile PBMC study, Bangladesh 450K study, and Bangladesh 850K study) adjusted for age, smoking status, cell type proportions, and sex (in the Chile studies). **C** and **D**. Volcano plot and Manhattan plot of meta-analysis of PBMC and buccal cell EWAS (i.e., the Chile PBMC study, Chile buccal cell study, Bangladesh 450K study, and Bangladesh 850K study) adjusted for age, smoking status, cell type proportions, and sex (in the Chile studies). In all plots, DVPs at *FDR* < 0.05 are shown as black points; the Bonferroni and FDR levels of significance are indicated by a solid and dashed red line, respectively; and DVRs are indicated by blue lines

Meta-analysis results of differential variability are summarized in Table 4. Significant probes are included in Table 5. Effects sizes and *p*-values of individual EWAS are included in Additional File 1 and measures of heterogeneity are listed in Supplemental Table S1. In our meta-analysis of PBMCs the genomic inflation factor did not show major departure from the expected distribution (λ = 1.17) (Table 4 and Supplemental Fig. S4). Although this genomic inflation factor was greater than that of our meta-analysis for DMPs, it has previously been shown that λ varies with the number of true associations [54]. We identified 23 DVPs (*FDR* < 0.05), of which 22 had a positive pulled effect size (Table 5 and Fig. 3A and B) and 21 had a positive effect estimate across all three EWAS (Additional File 1). Approximately half of FDR-significant DVPs showed evidence for heterogeneity (among 11 probes, *I*^*2*^ ≥ 58.2 and *p*_*heterogeneity*_ < 0.10) (Supplemental Table S1).

Our DVP PBMC + buccal cell meta-analysis had a λ = 1.18 and resulted in 19 DVPs (*FDR* < 0.05) (Table 4). Thirteen DVPs had a positive pooled effect size (Table 5 and Fig. 3A and B). Among significant DVPs, 15 CpGs had a consistent direction of association across all four EWAS (Additional File 1). Seven of the 19 DVPs showed evidence for heterogeneity (*I*^*2*^ ≥ 54.4 and *p*_*heterogeneity*_ < 0.10) (Supplemental Table S1).

We did not observe overlap between DMPs and DVPs Twelve DVPs were identified in both the PBMC meta-analysis and PBMC + buccal cell meta-analysis.

We identified 11 and 29 DVRs from PBMC meta-analysis results and PBMC + buccal cell meta-analysis results, respectively (Table 6 and Fig. 3B and D). Nine DVRs were common for both PBMC and PBMC + buccal cell analyses.

A total of 85 DVPs with *FDR* < 0.10 in our fully adjusted PBMC meta-analysis were included in KEGG pathway analyses. We identified five KEGG pathways containing an overrepresentation of differentially variable genes: N-glycan biosynthesis (*p* = 0.014), leishmaniasis (*p* = 0.036), endocytosis (*p* = 0.042), IL-17 signaling pathway (p = 0.042), and toll-like receptor signaling pathway (*p* = 0.050) (Fig. 2 and Supplemental Table S2). Among results from the PBMC + buccal cell meta-analysis, 106 probes (*FDR* < 0.10) were included in a pathways analysis, yielding four KEGG pathways: fructose and mannose metabolism (*p* = 0.014), arachidonic acid metabolism (*p* = 0.017), endocytosis (*p* = 0.017), and cell adhesion molecules (*p* = 0.049). AMPK signaling pathway was also among the top KEGG pathways but did not achieve statistical significance (*p* = 0.051). No pathways were significant after an FDR correction.

Based on review of published EWAS, none of our DMPs or DVPs had previously been associated with arsenic exposure. We also compared genes containing differential methylation to previous EWAS. The DMP cg20784693 (*HDAC4*) and the DVPs cg00355447 (*HCG18*), cg23281729 (*PARD6G*), cg07611790 (*RASIP1*), cg13490635 (*RBPMS*), cg11691429 (*SNTG2*), and cg22749736 (*TOLLIP*) are located in genes previously identified as including differentially methylated CpGs with total maternal urinary arsenic in cord blood in a birth cohort in Mexico (*FDR* < 0.05) [16] (summarized in Supplemental Table S3). *HNRNPUL1*, annotated to the DVP cg26384602, was also found to be differentially methylated with maternal urinary arsenic in cord blood in a Taiwanese birth cohort [17]. We also compared our DMPs and DVPs to all nominally significant DMPs previously associated with urinary arsenic levels among adults in the US (*p* < 0.05) [9]. Notably, *HDAC4* included 38 DMPs, *SNTG2* included 9 DMPs, and *TOLLIP* included 8 DMPs identified by Bozack et al. in the Strong Heart Study, a cohort of American Indian adults from the Great Plains and the Southwest [9] (*p* < 0.05).

*SLC39A4*, *WNT16*, and *GSE1*, located in DMRs in our meta-analyses; and *CELSR3*, *RUFY1*, and *TNXB*, located in DVRs, were found to include a differentially methylated CpGs in Rojas et al. In addition, *NKX2–6* and *SNAI3*, annotated to DMRs in our meta-analyses; and *ZNF875*, annotated to a DVR, have previously been identified as differentially methylated among adults with arsenicosis in Mexico [55]. Expression of the immune response gene *HLA-DRB1*, located in a DVR in our study, has been associated with arsenic exposure in a case-control study of adults in the US [56].

## Discussion

This study utilizes four DNAm datasets to analyze epigenome-wide associations with arsenic exposure using a two-step approach of (1) a harmonized data processing and analysis pipeline and (2) meta-analysis to combine individual EWAS results. We leverage data from two distinct populations with high chronic arsenic exposure: adults in Chile exposed to arsenic prenatally and early in life and adults in Bangladesh with concurrent high arsenic exposure. Although DNAm was measured in different tissue types (i.e., PBMCs and buccal cells in the Chile study) and using different platforms (i.e., the 850 K and 450 K microarrays), we identified positions and regions with significant differential methylation and variability (*FDR* < 0.05).

We conducted meta-analyses of differential methylation and differential variability limiting to PBMC EWAS and including both PBMC and buccal cell EWAS, identifying 1 and 3 DMPs, and 23 and 19 DVPs, respectively (*FDR* < 0.05). Although no DMPs overlapped between meta-analyses with and without buccal cells, 12 DVPs were common for both analyses. In addition, we identified 11 and 16 DMRs, and 11 and 19 DVRs limiting analyses to PBMCs and including buccal cells, respectively. Eight DMRs overlapped between analyses and 9 DVRs overlapped between analyses with and without including buccal cells.

We identified a greater number of statistically significant DVPs than DMPs. To our knowledge, this is the first study to investigate associations between chronic arsenic exposure and differential DNAm variability; however, results suggest the importance of differential variability as a biological mechanism or biomarker of arsenic exposure. Genomic regions with increased variability in methylation have been associated with functional control of gene expression (e.g., transcription start sites) [57, 58] and Gene Ontology pathways related to development [59]. Variably methylated regions may be particularly responsive to environmental conditions. For example, stochastic and environmentally induced DNAm variability could contribute to epigenetic drift not necessarily associated with mean changes in DNA methylation. The hypothesis that increased DNAm variability might precede neoplasia has been supported for different tissues [60, 61]. Environmental exposures have been shown to increase variability of DNAm. For example, a study of genetically-identical fibroblasts cultured under normal and low-nutrient conditions identified 135 regions containing differential variability and enrichment for imprinted genes [58].

Although we did not observe overlap between our results and previous EWAS on the level of individual loci, there was substantial overlap between annotated genes. Two of our DMPs and five DVPs were annotated to differentially methylated genes associated with maternal urinary arsenic in an EWAS of cord blood in Mexico (*FDR* < 0.05) [16], and two DMPs and 22 DVPs were annotated to genes associated with urinary arsenic in an EWAS of adults exposed to arsenic in the US (*p* < 0.05). We also observed overlap between genes located in our DMRs and DVRs and genes including differential methylation associated with prenatal and adult exposure [16, 55].

Lack of a consistent epigenetic signature of arsenic exposure across EWAS suggests that arsenic broadly impacts DNAm across the genome or that detected signals are influenced by population characteristics. However, arsenic may also be impacting epigenetic dysregulation on the level of common genes or regions, rather than specific loci. This might be supported by the observed increase in DNA methylation variability rather than mean changes to individual sites.

In pathway analyses, we identified several KEGG pathways with biological relevance to arsenic exposure. KEGG pathways associated with DMPs in our PBMC and PBMC + buccal cell meta-analyses included lysosome, autophagy, and mTOR signaling. The AMPK signaling pathway was among the top 5 associated with DVPs in PBMC and PBMC + buccal cell meta-analyses, although it did not achieve statistical significance (*p* = 0.051). Autophagy is regulated by mTORC1 and AMPK; mTORC1 represses autophagy whereas AMPK promotes autophagy [62]. Reactive oxygen species may affect autophagy by inhibiting mTORC1 [63]. Autophagy is a potential mechanism through which arsenic exposure induces adverse health outcomes including type 2 diabetes mellitus (T2DM); hepatic autophagy impacts cellular metabolism and affects glucagon and insulin levels [64]. In a mouse model with a high fat diet, arsenic exposure increased oxidative stress and hepatic autophagy, a potential mediating pathway between arsenic exposure and T2DM [63]. In addition, arsenic exposure may be related to lysosome activity as seen in treatment of acute promyelocytic leukemia (APL). An in vitro study demonstrated that arsenite destabilizes lysosomes, releasing proteases which act on the promyelocytic leukemia and retinoic acid receptor α (PML/RARα), a fusion protein expressed by APL cells [65].

The KEGG pathway one carbon pool by folate was also associated with DMPs in our PBMC and PBMC + buccal cell meta-analyses. One-carbon metabolism (OCM) is the biochemical pathway that synthesizes S-adenosylmethionine (SAM). SAM serves as the methyl donor in arsenic metabolism [66], a process that reduces arsenic toxicity and facilitates urinary excretion of arsenic. Although arsenic methylation capacity is influenced by one-carbon metabolism [67], it is not known the extent to which arsenic exposure may affect regulation of OCM. It should be noted, however, that results of KEGG pathway analyses are based on statistical associations and should be interpreted with caution. Further research is necessary to understand the relationships between arsenic exposure, changes in DNAm, and dysregulation of biological pathways.

This study has several limitations. We leveraged data from studies of two populations that differed in genetic background, exposure levels and measurement, and timing of exposure, and it is not known how these factors may impact associations between arsenic and epigenetic dysregulation. For example, changes in DNAm due to exposure limited to prenatal and early-life periods may not persist into adulthood. In addition, the Chile and Bangladesh studies may have exposure misclassification. In the Chile study, participants were classified as exposed or not exposed based on place of birth although good historical records of arsenic water concentration are available in the study area, and in the Bangladesh study, participants were classified based on well water arsenic concentration. However, we expect exposure misclassification to be nondifferential. Our choice of a water arsenic concentration cutoff to classify high and low exposure in the Bangladesh studies may have influenced results. Dichotomizing continuous variables is rarely warranted; however, we chose to use this dichotomous exposure variable defined by values at or near the median for each study (1) due to lack of continuous exposure data in the Chile studies, (2) to minimize exposure misclassification that could be introduced by a single-timepoint continuous variable representing chronic exposure throughout participants’ lifetimes, and (3) to ensure sufficient contrast between high- and low-exposure groups while maximizing group size. It should also be noted that although including multiple tissue types could increase heterogeneity across EWAS, a smaller proportion of FDR-significant DVPs identified in our PBMC + buccal cell meta-analysis had evidence of heterogeneity (seven of 19 DVPs had *I*^*2*^ ≥ 54.4 and *p*_*heterogeneity*_ < 0.10) than in our meta-analysis restricted to PBMCs (11 of 23 DVPs had *I*^*2*^ ≥ 58.2 and *p*_*heterogeneity*_ < 0.10). The observed heterogeneity may also be due in part to the number of studies included in our meta-analyses, as *I*^*2*^ may be biased in meta-analyses with few studies [68].

The Chile studies may also have been influenced by sex-specific effects. Our ability to test for sex-specific effects was limited by small samples sizes and DNAm was measured among males only in the Bangladesh studies. Although sex-specific associations between arsenic exposure and other measures of epigenetic regulation including global levels of 5-hydroxymethylcytosine [69], 5-methylcytosine [70], and post-translational histone modifications [7] have been reported, few EWAS of arsenic exposure have reported sex-stratified analyses. In an EWAS of cord blood DNAm in Bangladesh (*N* = 127), 3 CpGs were associated with arsenic exposure in early gestation among male infants only (*FDR* < 0.05), and there was a stronger trend toward a negative association between exposure and DNAm among males (74% of the top 500 CpGs among males vs. 41% among females) [12]. A greater number of DMPs was also identified among males in an EWAS of prenatal arsenic exposure on DNAm measured at age 9 in Bangladesh (*N* = 113): 9, 57, and 15 CpGs were associated with exposure among children overall, males, and females, respectively (*FDR* < 0.05), with no overlap between CpGs identified in sex-stratified analyses [14]. However, in an EWAS of arsenic exposure in the Strong Heart Study (*N* = 2,325), substantial sex-specific effects in stratified analyses were not observed with all FDR-significant CpGs achieving nominal significance in sex-stratified analyses (*p* < 0.05) [9]. Further, in our study, there is insufficient evidence to conclude that prenatal exposure is associated with sex in the Chile study’s datasets (Pearson’s chi-squared test *p* = 1, 0.88 in the PBMC and the buccal studies, respectively). However, considering previously observed sex-specific associations, future meta-analyses including larger samples sizes should investigate potential sex-specific effects through stratified analyses.

This study was strengthened by using a common data processing and analysis pipeline. Differences in data processing methods including statistical methods applied to minimize bias (e.g., normalization, correction for cell type distribution) may limit the ability to compare findings across previous EWAS. In addition, small study sizes and inadequate statistical power have been frequent limitations of previous EWAS of arsenic exposure [8]. Although the four individual EWAS used in this study had small sample sizes, combining results in a meta-analysis allowed us to increase statistical power and detect significant associations with exposure. As the first meta-analysis of DNAm and arsenic exposure, this study may serve a model for conducting a larger meta-analysis leveraging EWAS with larger samples sizes and inclusive of more diverse populations and exposure levels, or as a foundation for a validation study conducted in a larger cohort.

## Conclusions

This study provides a model for leveraging EWAS with small sample sizes to detect epigenome-wide associations with environmental exposures. To our knowledge, this is the first meta-analysis of associations between chronic arsenic exposure and DNAm and may provide a framework for conducting larger meta-analyses. Drawing upon four EWAS conducted in distinct populations (adults with high prenatal and early-life exposure in Chile and adults with high concurrent exposure in Bangladesh) and tissue types (PBMCs and buccal cells), we identified differential mean and variable methylation at individual loci and regions. We also identified KEGG pathways that may be related to mechanisms of arsenic toxicity. Future meta-analyses including studies conducted in different populations may provide more information regarding associations between chronic arsenic exposure and epigenetic dysregulation. In addition, research is needed to fully understand the downstream effects of differences in DNAm levels and variability on gene expression and health outcomes.

## Supplementary Information

**Additional file 1: Table 1**: Effect sizes and *p*-values from individual EWAS of differentially methylated and variable probes identified in meta-analyses at FDR < 0.05.

**Additional file 2: Fig. S1:** The leading principal components of the 5000 most variable CpG sites across the Chile study’s buccal cell and PBMC samples suggest that within-participant similarity is not a significant source of variability. **Fig. S2:** A permutation test of within-individual similarity scores identifies one nominally significant pair of samples (*p* < 0.05). After correcting for multiple testing (FDR < 0.05, FWER < 0.05), no samples are found to be significantly similar. **Fig. S3:** Q-Q plot of meta-analysis results of differentially methylated positions (DMPs) in PBMC EWAS (i.e., the Chile PBMC study, Bangladesh 450 K study, and Bangladesh 850 K study) adjusted for age, smoking status, cell type proportions, and sex in the Chile studies. **Supplemental Table S1:** Heterogeneity of differentially methylated positions (DMPs) and differentially variable positions (DVPs) in meta-analyses. DMPs and DVPs (*FDR* < 0.05) in the PBMC meta-analysis (i.e., including the Chile PBMC study, Bangladesh 450 K study, and Bangladesh 850 K study) and PBMC + buccal cell meta-analysis (i.e., including the Chile buccal cell study) adjusted for age, smoking status, cell type proportions, and sex (in the Chile studies). **Supplemental Table S2:** KEGG pathway analysis of differentially methylated regions (DMRs) and differentially variable regions (DVRs) in the PBMC meta-analysis (i.e., including the the Chile PBMC study, Bangladesh 450 K study, and Bangladesh 850 K study) and PBMC + buccal cell meta-analysis (i.e., including the Chile buccal cell study) adjusted for age, smoking status, cell type proportions, and sex (in the Chile studies). Pathways significant at *p* < 0.05 are shown. **Supplemental Table S3:** Summary of genes containing differentially methylated positions (DMPs) of differentially variable positions (DVPs) identified in meta-analyses in and previous EWAS.

## Data Availability

The datasets generated and/or analyzed during the current study are not publicly available due to participant confidentiality but are available from the corresponding author on reasonable request and upon institutional review board review. Code used in data processing and EWAS, and complete lists of differentially methylated positions (DMPs) and differentially variable positions (DVPs) (*p* < 0.05) in individual EWAS are available at the study’s Github repository (github.com/annebozack/SRP_arsenic_DNAm_metaanalysis).

## Abbreviations

DNAm: DNA methylation
EWAS: Epigenome-wide association study
450K: HumanMethylation BeadChip
850K: Infinium MethylationEPIC BeadChip
US: United States
PBMCs: peripheral blood mononuclear cells
DMPs: Differentially methylated positions
DVPs: Differentially variable positions
DMRs: Differentially methylated regions
DVRs: Differentially variable regions
IRB: Institutional Review Board
UC: University of California
PBS: Phosphate buffered saline
DMSO: Dimethyl sulfoxide
QB3: California Institute for Quantitative Biosciences
FACT: Folic Acid and Creatine Trial
HEALS: Health Effects of Arsenic Longitudinal Study
ICP-MS: Inductively coupled plasma mass spectrometry
FunNorm: Functional normalization
PCA: Principal component analysis
NK: Natural killer cells
FDR: False discovery rate
KEGG: Kyoto Encyclopedia of Genes and Genomes
T2DM: Type 2 diabetes mellitus
APL: Acute promyelocytic leukemia
PML/RARα: Promyelocytic leukemia and retinoic acid receptor α
SAM: S-adenosylmethionin
