# Supplementary material for: Exposure to arsenic at different life-stages and DNA methylation meta-analysis in buccal cells and leukocytes
Source: Environ Health. 2021 Jul 9;20:79. doi: 10.1186/s12940-021-00754-7 (PMC8272372; doi:10.1186/s12940-021-00754-7)
Supplement: Supplementary file 2 — Additional file 2: Fig. S1: The leading principal components of the 5000 most variable CpG sites across the Chile study’s buccal cell and PBMC samples suggest that within-participant similarity is not a significant source of variability. Fig. S2: A permutation test of within-individual similarity scores identifies one nominally significant pair of samples (p < 0.05). After correcting for multiple testing (FDR < 0.05, FWER < 0.05), no samples are found to be significantly similar. Fig. S3: Q-Q plot of meta-analysis results of differentially methylated positions (DMPs) in PBMC EWAS (i.e., the Chile PBMC study, Bangladesh 450 K study, and Bangladesh 850 K study) adjusted for age, smoking status, cell type proportions, and sex in the Chile studies. Supplemental Table S1: Heterogeneity of differentially methylated positions (DMPs) and differentially variable positions (DVPs) in meta-analyses. DMPs and DVPs (FDR < 0.05) in the PBMC meta-analysis (i.e., including the Chile PBMC study, Bangladesh 450 K study, and Bangladesh 850 K study) and PBMC + buccal cell meta-analysis (i.e., including the Chile buccal cell study) adjusted for age, smoking status, cell type proportions, and sex (in the Chile studies). Supplemental Table S2: KEGG pathway analysis of differentially methylated regions (DMRs) and differentially variable regions (DVRs) in the PBMC meta-analysis (i.e., including the the Chile PBMC study, Bangladesh 450 K study, and Bangladesh 850 K study) and PBMC + buccal cell meta-analysis (i.e., including the Chile buccal cell study) adjusted for age, smoking status, cell type proportions, and sex (in the Chile studies). Pathways significant at p < 0.05 are shown. Supplemental Table S3: Summary of genes containing differentially methylated positions (DMPs) of differentially variable positions (DVPs) identified in meta-analyses in and previous EWAS. [file 12940_2021_754_MOESM2_ESM.docx]

**Exposure to arsenic at different life-stages and DNA methylation meta-analysis in buccal cells and leukocytes**

**Authors:** Anne K. Bozack,^1^ Philippe Boileau,^2^ Linqing Wei,^2^ Alan E. Hubbard,^2^ Fenna C. M. [Sillé](https://pubmed.ncbi.nlm.nih.gov/?term=Sill%C3%A9+FCM&cauthor_id=30708400),^3^ Catterina Ferreccio,^4^ Johanna Acevedo,^5,6^ Lifang Hou,^7^ Vesna Ilievski,^8^ Craig M. Steinmaus,^1^ Martyn T. Smith,^1^ Ana Navas-Acien,^8^ Mary V. Gamble,^8^ and Andres Cardenas^1^

**SUPPLEMENTAL MATERIALS**

**Figure S1:** The leading principal components of the 5,000 most variable CpG sites across the Chile study’s buccal cell and PBMC samples suggest that within-participant similarity is not a significant source of variability.

**
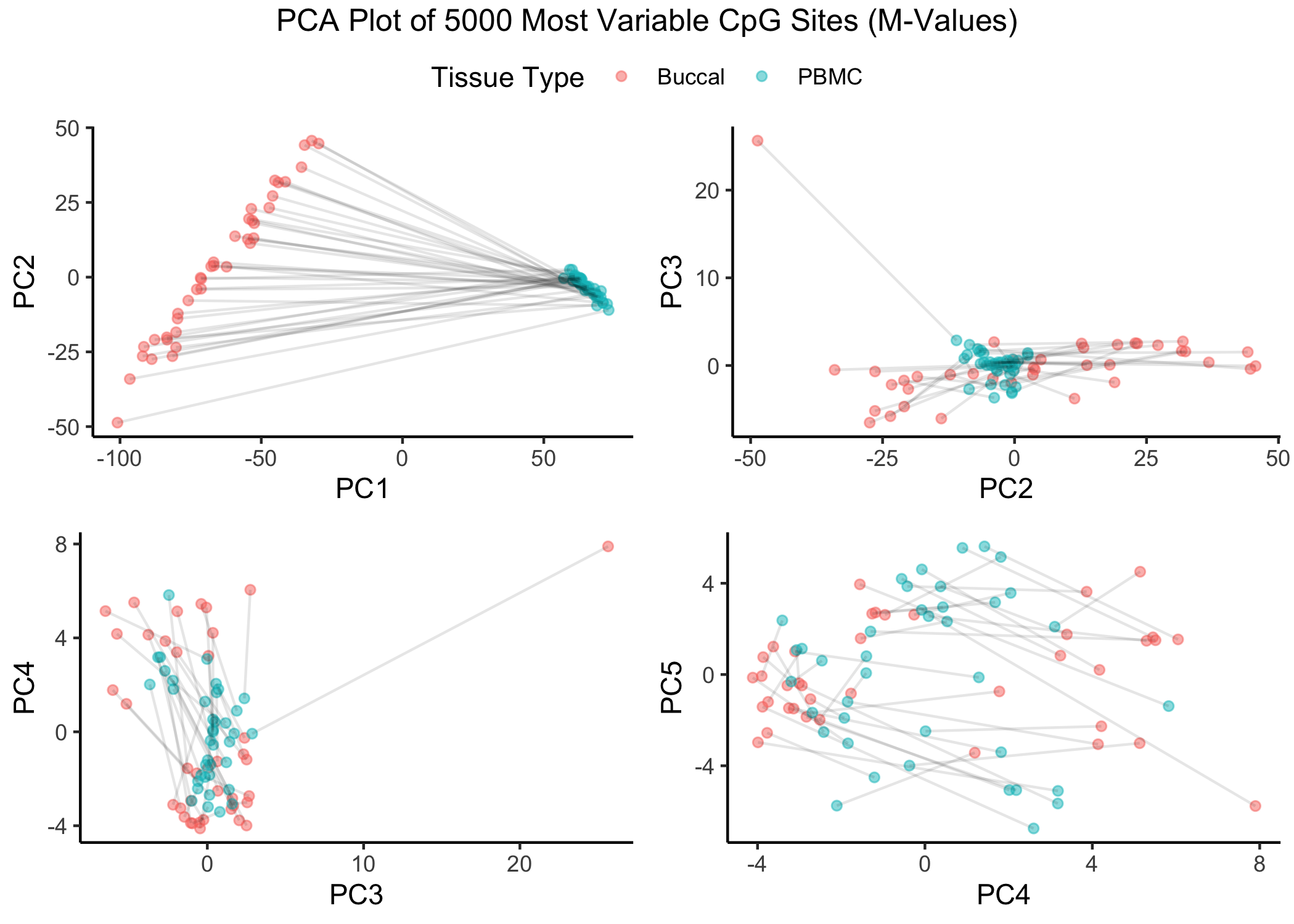
**

**Figure S2:** A permutation test of within-individual similarity scores identifies one nominally significant pair of samples (p < 0.05). After correcting for multiple testing (FDR < 0.05, FWER < 0.05), no samples are found to be significantly similar.

**
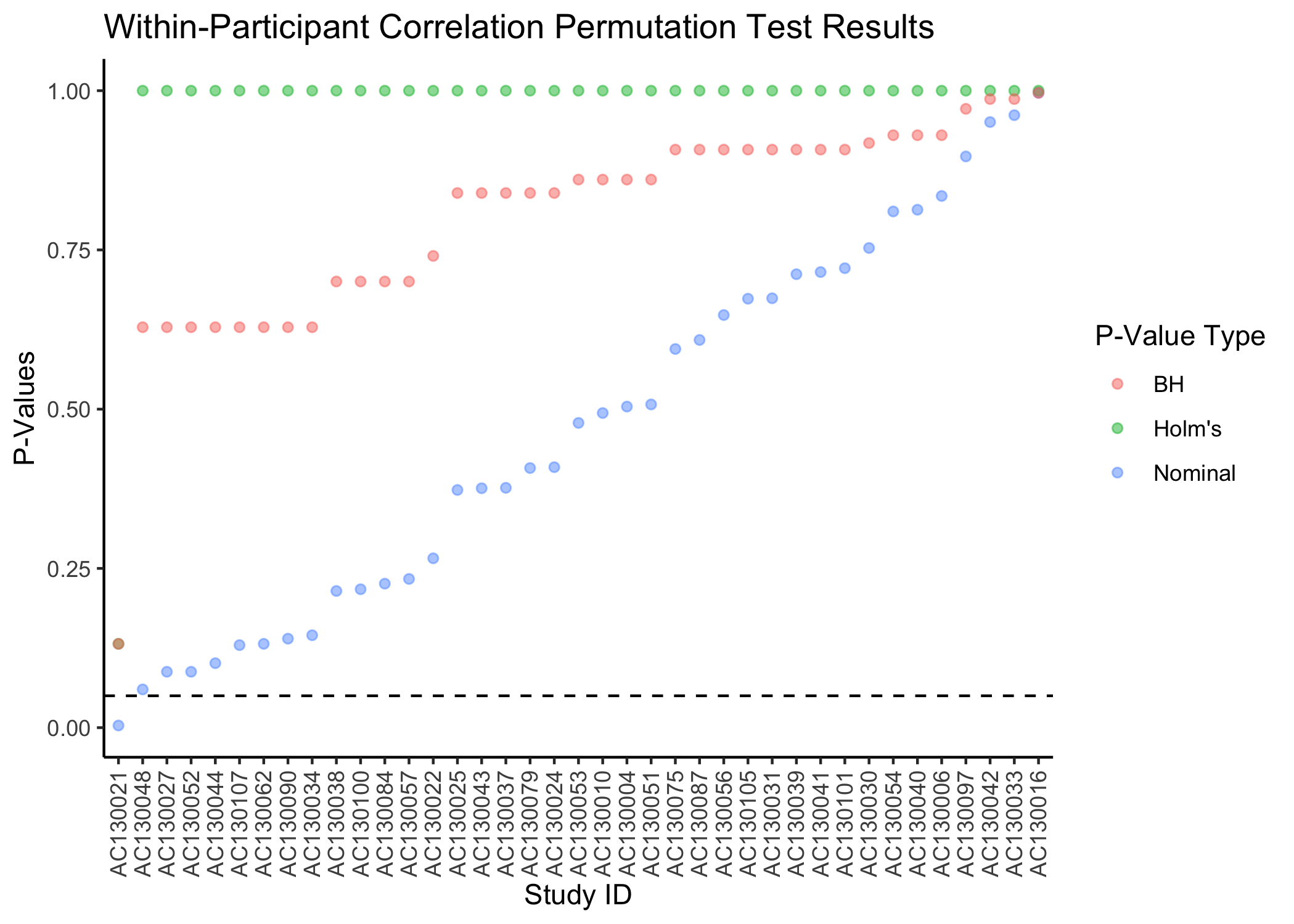
**

**Figure S3:** Q-Q plot of meta-analysis results of differentially methylated positions (DMPs) in PBMC EWAS (i.e., the Chile PBMC study, Bangladesh 450K study, and Bangladesh 850K study) adjusted for age, smoking status, cell type proportions, and sex in the Chile studies.

**
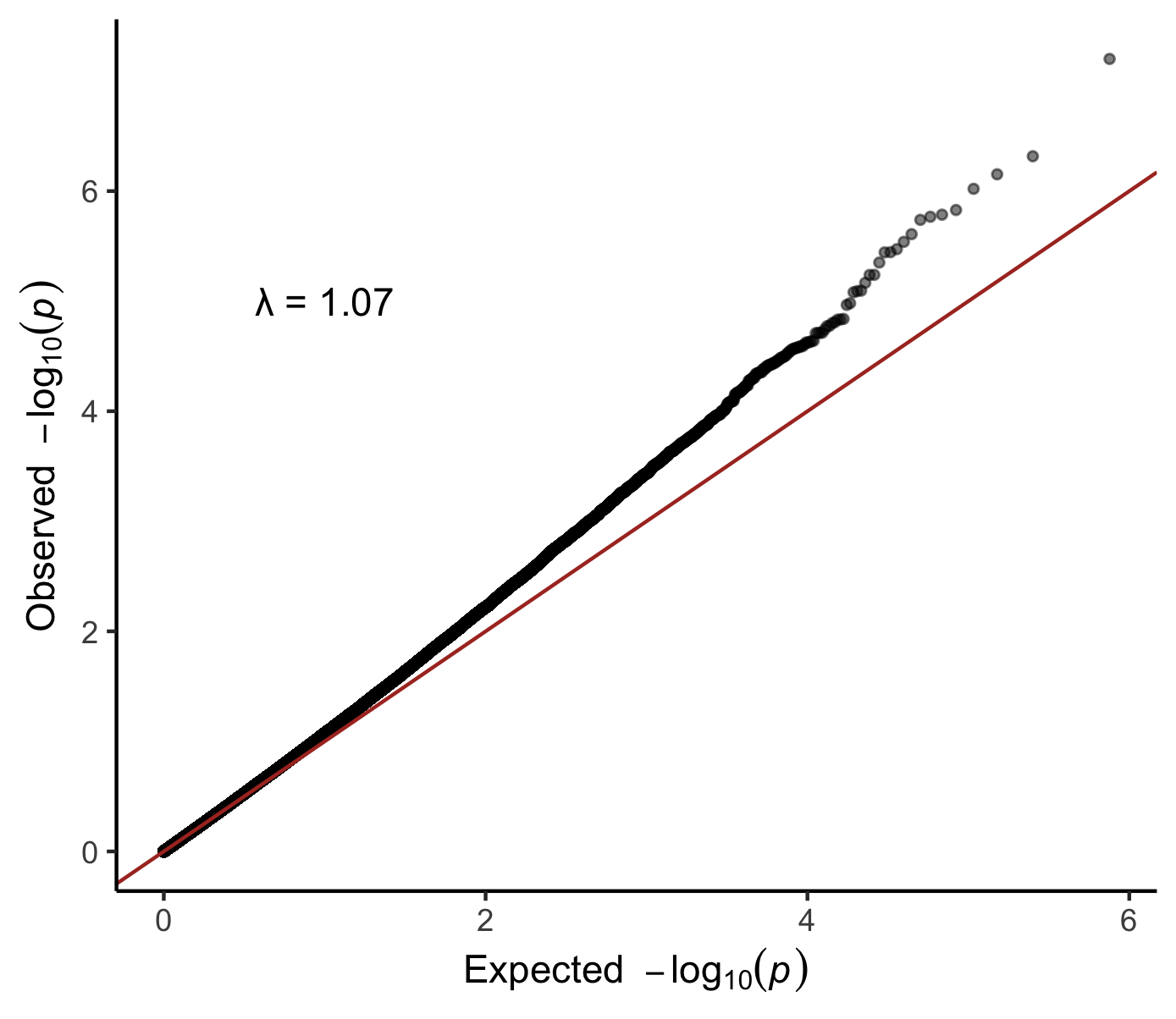
**

**Figure S4:** Q-Q plot of meta-analysis results of differentially variable positions (DVPs) in PBMC EWAS (i.e., the Chile PBMC study, Bangladesh 450K study, and Bangladesh 850K study) adjusted for age, smoking status, cell type proportions, and sex in the Chile studies.


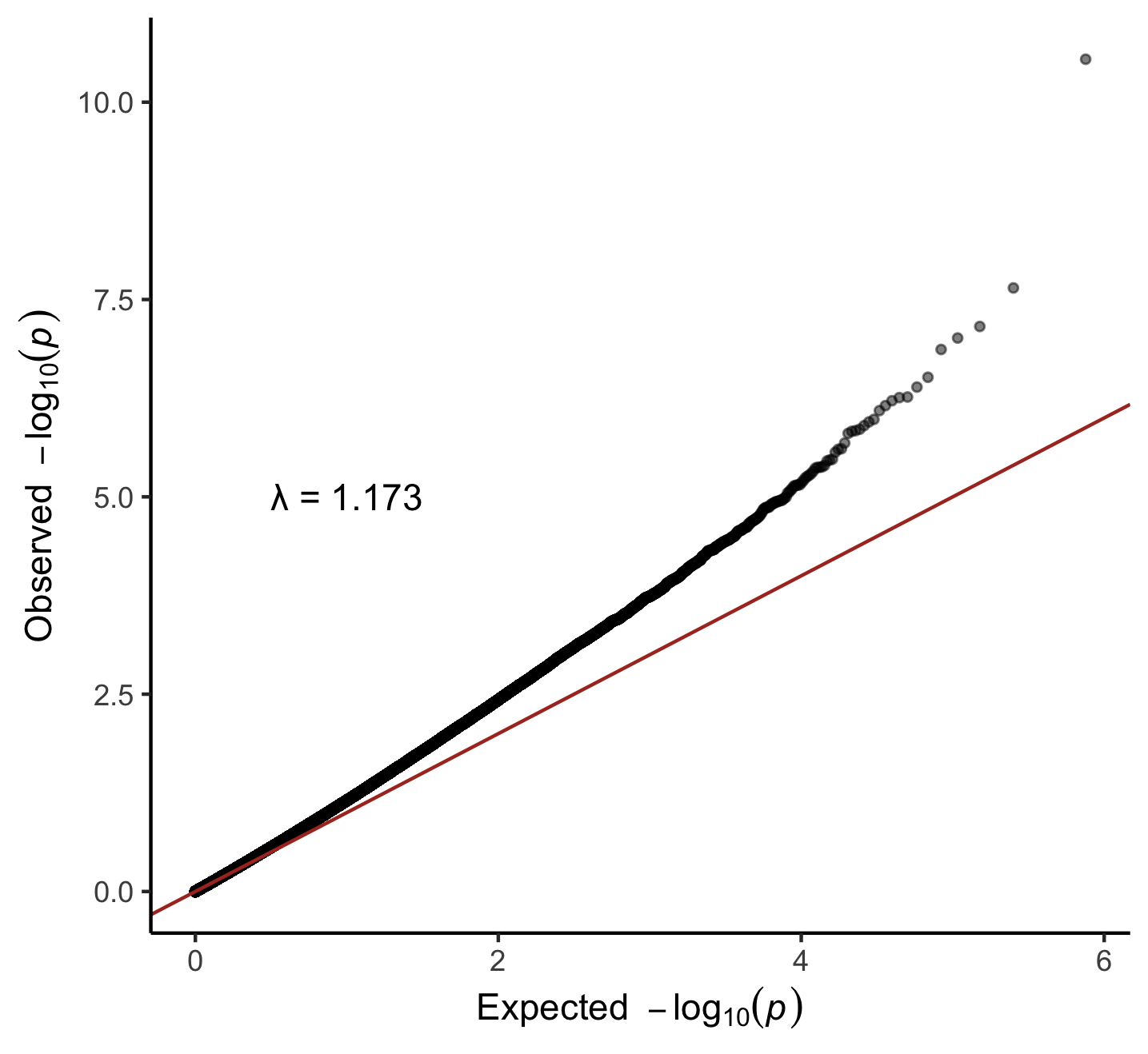


**Supplemental Table S1:** Heterogeneity of differentially methylated positions (DMPs) and differentially variable positions (DVPs) in meta-analyses. DMPs and DVPs (*FDR* < 0.05) in the PBMC meta-analysis (i.e., including the Chile PBMC study, Bangladesh 450K study, and Bangladesh 850K study) and PBMC + buccal cell meta-analysis (i.e., including the Chile buccal cell study) adjusted for age, smoking status, cell type proportions, and sex (in the Chile studies).

| **CpG** | **Effect** | **Direction ^a^** | ***p*** | ***FDR*** | ***I^2^*** | ***p_heterogeneity_^b^*** |
| --- | --- | --- | --- | --- | --- | --- |
| **DMPs** | | | | | | |
| **PBMCs** | | | | | | |
| cg13490635 | 0.31 | + + + | 6.30e-08 | 0.024 | 43.6 | 0.17 |
| **PBMCs + buccal cells** | | | | | | |
| cg09275980 | 0.31 | + + + + | 2.52e-07 | 0.042 | 68.4 | 0.023 |
| cg20784693 | -0.64 | - - - - | 3.08e-07 | 0.042 | 0.0 | 0.47 |
| cg18263451 | -0.12 | - - + - | 3.31e-07 | 0.042 | 77.3 | 0.004 |
| **DVPs** | | | | | | |
| **PBMCs** | | | | | | |
| **cg23281729** | 0.97 | - + + | 2.86E-11 | 1.08E-05 | 84.7 | 0.001 |
| **cg27425262** | 0.82 | + + + | 2.26E-08 | 0.004 | 78.0 | 0.011 |
| **cg11361658** | 0.17 | + + + | 6.94E-08 | 0.009 | 2.2 | 0.36 |
| **cg03205258** | 0.69 | + + + | 9.72E-08 | 0.009 | 58.2 | 0.09 |
| **cg09692492** | -0.35 | - - - | 1.36E-07 | 0.010 | 42.3 | 0.18 |
| **cg11032634** | 0.77 | + + + | 3.06E-07 | 0.019 | 61.8 | 0.07 |
| **cg16511076** | 0.13 | + + + | 4.07E-07 | 0.022 | 0.0 | 0.79 |
| **cg22387890** | 0.55 | + + + | 5.44E-07 | 0.023 | 66.8 | 0.049 |
| **cg22749736** | 0.13 | + + + | 5.53E-07 | 0.023 | 6.2 | 0.34 |
| **cg04080724** | 0.14 | + + + | 6.06E-07 | 0.023 | 0.0 | 0.47 |
| **cg05169951** | 0.23 | + + + | 6.99E-07 | 0.024 | 4.9 | 0.35 |
| cg00668103 | 0.14 | + + + | 8.08E-07 | 0.025 | 0.0 | 0.86 |
| cg08207566 | 0.12 | + + + | 1.04E-06 | 0.030 | 61.9 | 0.07 |
| cg01101647 | 0.13 | + + + | 1.13E-06 | 0.030 | 79.6 | 0.007 |
| cg07611790 | 0.11 | + + + | 1.25E-06 | 0.031 | 0.0 | 0.86 |
| cg11691429 | 0.21 | + + + | 1.39E-06 | 0.031 | 12.7 | 0.32 |
| **cg21185289** | 0.38 | + + + | 1.45E-06 | 0.031 | 0.0 | 0.93 |
| cg08285768 | 0.11 | + + + | 1.48E-06 | 0.031 | 62 | 0.07 |
| cg13972353 | 0.15 | + + + | 1.57E-06 | 0.031 | 0.0 | 0.69 |
| cg06740986 | 0.11 | + + + | 2.08E-06 | 0.039 | 0.0 | 0.37 |
| cg21857098 | 0.12 | + + + | 2.46E-06 | 0.043 | 68.6 | 0.041 |
| cg03479942 | 0.13 | + + + | 2.50E-06 | 0.043 | 65.5 | 0.06 |
| cg07984508 | 0.11 | + + + | 2.73E-06 | 0.045 | 80.6 | 0.006 |
| **PBMCs + buccal cells** | | | | | | |
| **cg23281729** | 0.99 | -+++ | 2.00E-14 | 7.51E-09 | 77.2 | 0.004 |
| **cg27425262** | 0.90 | + + + + | 4.52E-13 | 8.53E-08 | 69.7 | 0.020 |
| **cg03205258** | 0.64 | + + + + | 7.95E-09 | 0.001 | 42.4 | 0.16 |
| **cg11032634** | 0.70 | + + + + | 5.69E-08 | 0.006 | 50.9 | 0.11 |
| **cg04080724** | 0.14 | + + + + | 1.14E-07 | 0.009 | 0.0 | 0.68 |
| cg26384602 | -0.12 | - - - - | 1.87E-07 | 0.010 | 71.8 | 0.014 |
| **cg11361658** | 0.16 | + + + + | 1.90E-07 | 0.010 | 29.8 | 0.23 |
| **cg09692492** | -0.30 | - - - - | 2.32E-07 | 0.011 | 49.7 | 0.11 |
| **cg22749736** | 0.12 | + + + + | 4.20E-07 | 0.018 | 11.6 | 0.34 |
| cg21921619 | -0.15 | - + - - | 5.48E-07 | 0.021 | 82.0 | 0.001 |
| cg07935287 | -0.13 | - - - - | 7.91E-07 | 0.026 | 54.4 | 0.09 |
| **cg05169951** | 0.22 | + + + + | 8.19E-07 | 0.026 | 0.0 | 0.40 |
| **cg21185289** | 0.37 | + + + + | 1.01E-06 | 0.030 | 0.0 | 0.95 |
| **cg22387890** | 0.48 | + + + + | 1.12E-06 | 0.030 | 63.4 | 0.042 |
| cg13171197 | 0.12 | + - + + | 1.46E-06 | 0.037 | 48.9 | 0.12 |
| cg24754507 | -0.10 | - - - - | 1.55E-06 | 0.037 | 23.7 | 0.27 |
| cg00355447 | 0.14 | + + + + | 1.80E-06 | 0.039 | 3.8 | 0.37 |
| **cg16511076** | 0.12 | + + + - | 1.87E-06 | 0.039 | 58.7 | 0.06 |
| cg04873963 | -0.10 | - - - - | 2.46E-06 | 0.049 | 41.3 | 0.16 |
| DMP: differentially methylated position; DVP: differentially variable position. a. Direction of association between arsenic exposure and DNAm in each EWAS. For PBMCs, associations are listed in the following order: Bangladesh 850K, Bangladesh 450K, Chile PBMCs. For PBMCs + buccal cells, associations are listed in the following order Bangladesh 850K, Bangladesh 450K, Chile PBMCs, Chile buccal cells. b. Cochran’s *Q* test. | | | | | | |

**Supplemental Table S2:** KEGG pathway analysis of differentially methylated regions (DMRs) and differentially variable regions (DVRs) in the PBMC meta-analysis (i.e., including the the Chile PBMC study, Bangladesh 450K study, and Bangladesh 850K study) and PBMC + buccal cell meta-analysis (i.e., including the Chile buccal cell study) adjusted for age, smoking status, cell type proportions, and sex (in the Chile studies). Pathways significant at *p* < 0.05 are shown.

| **Pathway** | **Description** | **N genes in pathway** | **N genes containing DMPs or DVPs** | ***p*** |
| --- | --- | --- | --- | --- |
| **DMPs** |  |  |  |  |
| **PBMCs** |  |  |  |  |
| hsa00062 | Fatty acid elongation | 25 | 1 | 0.012 |
| hsa01212 | Fatty acid metabolism | 54 | 1 | 0.030 |
| hsa04142 | Lysosome | 123 | 1 | 0.047 |
| **PBMCs + buccal cells** | |  |  |  |
|  |  |  |  |  |
| hsa00670 | One carbon pool by folate | 19 | 1 | 0.019 |
| hsa04964 | Proximal tubule bicarbonate reclamation | 21 | 1 | 0.023 |
| hsa04150 | mTOR signaling pathway | 150 | 2 | 0.028 |
| hsa00062 | Fatty acid elongation | 25 | 1 | 0.035 |
| hsa04136 | Autophagy - other | 29 | 1 | 0.042 |
| **DVPs** |  |  |  |  |
| **PBMCs** |  |  |  |  |
| hsa00513 | Various types of N-glycan biosynthesis | 37 | 2 | 0.014 |
| hsa05140 | Leishmaniasis | 65 | 2 | 0.036 |
| hsa04144 | Endocytosis | 238 | 4 | 0.042 |
| hsa04657 | IL-17 signaling pathway | 87 | 2 | 0.042 |
| hsa04620 | Toll-like receptor signaling pathway | 83 | 2 | 0.050 |
| **PBMCs + buccal cells** | |  |  |  |
| hsa00051 | Fructose and mannose metabolism | 32 | 2 | 0.014 |
| hsa00590 | Arachidonic acid metabolism | 61 | 2 | 0.017 |
| hsa04144 | Endocytosis | 238 | 5 | 0.017 |
| hsa04514 | Cell adhesion molecules | 133 | 3 | 0.049 |

**Supplemental Table S3:** Summary of genes containing differentially methylated positions (DMPs) of differentially variable positions (DVPs) identified in meta-analyses in and previous EWAS.

| **CpG** | **Gene** | **Associations in meta-analyses** | **Previously identified associations annotated to gene** |
| --- | --- | --- | --- |
| cg13490635 | *RBPMS* | DMP in PBMC meta-analysis | 1 CpG (*FDR* < 0.05; Rojas et al. 2015)  1 CpG (*p* < 0.05; Bozack et al. 2020) |
| cg20784693 | *HDAC4* | DMP in PBMC + buccal cell meta-analysis | 4 CpGs (*FDR* < 0.05; Rojas et al. 2015)  38 CpGs (*p* < 0.05; Bozack et al. 2020) |
| cg23281729 | *PARD6G* | DVP in PBMC and PBMC + buccal cell meta-analyses | 1 CpG (*FDR* < 0.05; Rojas et al. 2015)  2 CpGs (*p* < 0.05; Bozack et al. 2020) |
| cg03205258; cg11032634 | *TXNRD2* | DVP in PBMC and PBMC + buccal cell meta-analyses | 3 CpGs (*p* < 0.05; Bozack et al. 2020) |
|  | *COMT* |  | 4 CpGs (*p* < 0.05; Bozack et al. 2020) |
| cg22387890 | *USP42* | DVP in PBMC and PBMC + buccal cell meta-analyses | 5 CpGs (*p* < 0.05; Bozack et al. 2020) |
| cg22749736 | *TOLLIP* | DVP in PBMC and PBMC + buccal cell meta-analyses | 2 CpGs (*FDR* < 0.05; Rojas et al. 2015)  8 CpGs (*p* < 0.05; Bozack et al. 2020) |
| cg04080724 | *C4orf49* | DVP in PBMC and PBMC + buccal cell meta-analyses | 1 CpG (*p* < 0.05; Bozack et al. 2020) |
| cg05169951 | *KREMEN2* | DVP in PBMC and PBMC + buccal cell meta-analyses | 1 CpG (*p* < 0.05; Bozack et al. 2020) |
|  | *PAQR4* |  | 2 CpGs (*p* < 0.05; Bozack et al. 2020) |
| cg00668103 | *C7orf51* | DVP in PBMC meta-analysis | 1 CpG (*p* < 0.05; Bozack et al. 2020) |
| cg01101647 | *EPHA5* | DVP in PBMC meta-analysis | 7 CpGs (*p* < 0.05; Bozack et al. 2020) |
| cg07611790 | *RASIP1* | DVP in PBMC meta-analysis | 1 CpG (*FDR* < 0.05; Rojas et al. 2015)  1 CpG (*p* < 0.05; Bozack et al. 2020) |
| cg11691429 | *SNTG2* | DVP in PBMC meta-analysis | 3 CpGs (*FDR* < 0.05; Rojas et al. 2015)  9 CpGs (*p* < 0.05; Bozack et al. 2020) |
| cg21185289 | *TLX2* | DVP in PBMC and PBMC + buccal cell meta-analyses | 1 CpG (*p* < 0.05; Bozack et al. 2020) |
| cg08285768 | *AKAP13* | DVP in PBMC meta-analysis | 11 CpGs (*p* < 0.05; Bozack et al. 2020) |
| cg13972353 | *SEC16A* | DVP in PBMC meta-analysis | 3 CpGs (*p* < 0.05; Bozack et al. 2020) |
| cg06740986 | *FAM129A* | DVP in PBMC meta-analysis | 1 CpG (*p* < 0.05; Bozack et al. 2020) |
| cg03479942 | *SNTB2* | DVP in PBMC meta-analysis | 1 CpG (*p* < 0.05; Bozack et al. 2020) |
| cg26384602 | *HNRNPUL1* | DVP in PBMC + buccal cell meta-analysis | 1 CpG (FDR < 0.05; Kaushal et al. 2017)  3 CpGs (*p* < 0.05; Bozack et al. 2020) |
| cg21921619 | *ATRNL1* | DVP in PBMC + buccal cell meta-analysis | 1 CpG (*p* < 0.05; Bozack et al. 2020) |
| cg07935287 | *WDR7* | DVP in PBMC + buccal cell meta-analysis | 1 CpG (*p* < 0.05; Bozack et al. 2020) |
| cg24754507 | *GPX6* | DVP in PBMC + buccal cell meta-analysis | 1 CpG (*p* < 0.05; Bozack et al. 2020) |
| cg00355447 | *HCG18* | DVP in PBMC + buccal cell meta-analysis | 6 CpGs (*FDR* < 0.05; Rojas et al. 2015)  4 CpGs (*p* < 0.05; Bozack et al. 2020) |
